# Metabolomic Approach to Identify Potential Biomarkers in KRAS-Mutant Pancreatic Cancer Cells

**DOI:** 10.3390/biomedicines12040865

**Published:** 2024-04-15

**Authors:** Boyun Kim, Jewon Jung

**Affiliations:** Department of SmartBio, College of Life and Health Science, Kyungsung University, Busan 48434, Republic of Korea; boyunism@gmail.com

**Keywords:** KRAS, TRPML1, pancreatic cancer, metabolomics, metabolite, biomarker

## Abstract

Pancreatic cancer is characterized by its high mortality rate and limited treatment options, often driven by oncogenic RAS mutations. In this study, we investigated the metabolomic profiles of pancreatic cancer cells based on their KRAS genetic status. Utilizing both KRAS-wildtype BxPC3 and KRAS-mutant PANC1 cell lines, we identified 195 metabolites differentially altered by KRAS status through untargeted metabolomics. Principal component analysis and hierarchical condition trees revealed distinct separation between KRAS-wildtype and KRAS-mutant cells. Metabolite set enrichment analysis highlighted significant pathways such as homocysteine degradation and taurine and hypotaurine metabolism. Additionally, lipid enrichment analysis identified pathways including fatty acyl glycosides and sphingoid bases. Mapping of identified metabolites to KEGG pathways identified nine significant metabolic pathways associated with KRAS status, indicating diverse metabolic alterations in pancreatic cancer cells. Furthermore, we explored the impact of TRPML1 inhibition on the metabolomic profile of KRAS-mutant pancreatic cancer cells. TRPML1 inhibition using ML-SI1 significantly altered the metabolomic profile, leading to distinct separation between vehicle-treated and ML-SI1-treated PANC1 cells. Metabolite set enrichment analysis revealed enriched pathways such as arginine and proline metabolism, and mapping to KEGG pathways identified 17 significant metabolic pathways associated with TRPML1 inhibition. Interestingly, some metabolites identified in PANC1 compared to BxPC3 were oppositely regulated by TRPML1 inhibition, suggesting their potential as biomarkers for KRAS-mutant cancer cells. Overall, our findings shed light on the distinct metabolite changes induced by both KRAS status and TRPML1 inhibition in pancreatic cancer cells, providing insights into potential therapeutic targets and biomarkers for this deadly disease.

## 1. Introduction

Pancreatic cancer ranks as the third most common cause of cancer-related deaths, with its mortality rate increasing by 0.3% annually. The features of this condition encompass inconspicuous symptoms, challenging early detection, limited survival duration, and unfavorable prognosis [1,2]. Its poor prognosis is highlighted by the strong link between disease occurrence and death rate [1]. In the United States, patients with pancreatic cancer continue to face a dismal five-year survival rate of merely 6%, primarily due to delayed diagnosis at the advanced and incurable stages, which emerges as the foremost contributing factor among various others [3]. Owing to the challenges in detecting the disease until its advanced stages, a substantial number of patients endure considerable suffering. While up to 20% of patients may qualify for initial resection, the prospects post-surgery are still desolate [3]. Despite undergoing potential curative resection, a majority of patients inevitably experience recurrence, with the five-year survival rate plateauing at a mere 25% [3,4]. To address this critical issue and enhance treatment outcomes, there is an urgent need to unravel the molecular mechanisms underlying early recurrence and chemoresistance in pancreatic cancer. Additionally, early diagnosis methods to detect warning signs should be developed. By gaining insights into these mechanisms, we can pave the way for more effective chemotherapy strategies and ultimately improve patient survival rates.

Pancreatic ductal adenocarcinoma (PDAC) constitutes the majority, approximately 85%, of pancreatic cancers, arising from the malignant transformation of ductal epithelial cells [5]. Genetically, PDAC is characterized by mutations in oncogenes or tumor suppressor genes, which play pivotal roles in disease progression. Notably, the frequencies of KRAS and TP53 mutations are approximately 85% and 60–70%, respectively [5,6,7]. Genetic analyses of clinical specimens have pinpointed KRAS mutations as early events, evident in stage 1 pancreatic intraepithelial neoplasia, while TP53 mutations are linked to the progression and invasiveness of pancreatic intraepithelial neoplasia at stage 3 [8]. The high incidence of KRAS mutations underscores the potential efficacy of targeted therapies against the RAS signaling network as a treatment strategy for PDAC [8]. However, despite extensive efforts, these therapeutic endeavors have yielded limited clinical benefits [8]. Notably, KRAS mutation has been associated with poorer survival rates and resistance to chemotherapy [9,10,11,12]. Given the prevalence of KRAS mutation in pancreatic cancer, it is imperative to explore alternative therapeutic approaches that may yield better responses [5,8]. Furthermore, the predominance of specific KRAS mutations, such as G12D and G12V, in PDAC underscores the importance of utilizing appropriate cell lines for research purposes. In this context, our study employs BxPC3 cells (KRAS-wildtype (WT); TP53 mutant) and PANC1 cells (KRAS-mutant G12D; TP53 mutant) to elucidate the mechanisms underlying pancreatic cancer chemoresistance and identify potential therapeutic targets. 

In parallel, advancements in high-throughput analyses, including genomics, transcriptomics, and proteomics, have identified potential biomarkers for pancreatic cancer [13]. However, there remains a notable gap in clinically relevant biomarkers for precise early-stage diagnosis [14]. Efforts to uncover novel markers with enhanced sensitivity and specificity are imperative to improving diagnostic accuracy and patient outcomes [15,16]. Integration of multi-omics data and the exploration of emerging technologies hold promise in further elucidating the intricate molecular landscape of pancreatic cancer [17,18]. Among these approaches, metabolomics stands out due to its ability to directly reflect underlying biochemical processes through metabolite analysis [19]. Thus, this study utilized untargeted metabolomic analysis to identify potential biomarkers and elucidate metabolic changes between KRAS-wildtype and -mutant pancreatic cancer cells to gain insights into overcoming treatment challenges in pancreatic cancer management.

## 2. Materials and Methods

### 2.1. Cell Culture

Pancreatic cancer cells, including KRAS-wildtype BxPC-3 and KRAS-mutant PANC1, were obtained from the Korean Cell Line Bank (KCLB). BxPC-3 cells were cultured in RPMI-1640 medium (Welgene, Gyeongsan-si, Republic of Korea), while PANC1 cells were cultured in DMEM medium (Welgene, Republic of Korea), both supplemented with essential nutrients including 10% FBS (HyClone, Logan, UT, USA), 4.5 g/L D-glucose, 2 mM L-glutamine, 1 mM sodium pyruvate, 1.5 g/L sodium bicarbonate, 100 U/mL penicillin, and 100 g/mL streptomycin (Thermo Fisher Scientific, Waltham, MA, USA). The cell cultures were maintained under standard conditions at 37 °C in a humidified atmosphere containing 5% CO_2_.

### 2.2. Cytotoxicity Assay

To assess cell viability, the 3-(4,5-dimethylthiazol-2-yl)-2,5-diphenyltetrazolium bromide (MTT) assay was utilized. Pancreatic cancer cells were treated with ML-SI1 (Sigma Aldrich, St. Louis, MO, USA) for 48 h and then exposed to 2 mg/mL MTT solution at 37 °C for 3 h in the dark. Upon completion of the incubation period, dimethyl sulfoxide (DMSO) was added to dissolve the formazan crystals generated by viable cells. The absorbance was subsequently measured at 540 nm using a microplate reader (SpectraMax^®^ ABS, Molecular Devices, San Jose, CA, USA).

### 2.3. Quantitative Real-Time Polymerase Chain Reaction (qRT-PCR)

Total RNA was extracted from BxPC3 and PANC1 cells using TRIzol reagent (Ambion, Life Technologies, Carlsbad, CA, USA) according to the manufacturer’s instructions. Subsequently, reverse transcription was carried out with 500 ng of total RNA using a High-Capacity cDNA RT kit (Applied Biosystems, Thermo Fisher Scientific) on a Bio-Rad T100 thermal cycler (Bio-Rad, Hercules, CA, USA). This step facilitated the conversion of RNA into complementary DNA (cDNA), enabling subsequent gene expression analysis. SYBR green-based qRT-PCR was conducted on a QuantStudioTM 3 Real-Time PCR System (Applied Biosystems, USA) to quantitatively measure the expression levels of target genes. Specifically, the expression of *MCOLN1* (NM_020533.3) and *TFEB* (NM_001167827.3) was analyzed using gene-specific primer pairs: MCOLN1 Forward: 5′-TCTTCCAGCACGGAGACAAC-3′, Reverse: 5′-GCCACATGAACCCCACAAAC-3′; TFEB Forward: 5′-CCAGAAGCGAGAGCTCACAGAT-3′, Reverse: 5′-TGTGATTGTCTTTCTTCTGCCG-3′. To ensure accurate normalization and reliable interpretation of gene expression data, the relative mRNA abundance was normalized to the expression of the reference gene *GAPDH* (NM_001256799.3). *GAPDH* was amplified using the following primer pair: *GAPDH* Forward: 5′-GAAGGTGAAGGTCGGAGTC-3′, Reverse: 5′-GAAGATGGTGATGGGATTTC-3′.

### 2.4. Sample Preparation for UHPLC/Q-TOF-MS

Untargeted metabolites from BxPC3 and PANC1 cells extracted using an ice-cold solution composed of 40% (*v*/*v*) acetonitrile, 40% (*v*/*v*) methanol, and 20% (*v*/*v*) H_2_O (extraction solvent). The collected pancreatic cancer cells were washed three times with ice-cold PBS, and the cells were rapidly snap-frozen using liquid nitrogen to preserve their metabolomic profile. For cell lysis and metabolite extraction, the snap-frozen cells were resuspended in the extraction solvent and transferred to new microcentrifuge tubes. To facilitate cell lysis and metabolite release, ultrasonication was performed in an ultrasonic bath. The ultrasonication protocol consisted of 30 s bursts of sonication followed by a 30 s rest period on ice, repeated for three cycles over a total duration of 3 min. This method ensured efficient disruption of cellular structures while minimizing sample heating. After ultrasonication, the samples were incubated on ice for 10 min to further facilitate metabolite extraction. Subsequently, the lysed cell suspensions were centrifuged at 12,000× *g* for 10 min at 4 °C to separate the cellular debris and intact organelles from the liquid phase containing the extracted metabolites. After centrifugation, the supernatant containing the extracted metabolites was carefully transferred to a new screw-cap glass tube with an insert (Agilent Technologies, Santa Clara, CA, USA) to minimize sample loss and contamination. Prior to UHPLC/Q-TOF-MS analysis, the liquid phase of each sample was filtered using a 0.22 μm microfiltration membrane to remove any remaining particulate matter that could interfere with the chromatographic separation and mass spectrometric detection.

### 2.5. Liquid Chromatography and Mass Spectrometry

Untargeted metabolite analysis was carried out using a liquid chromatograph quadrupole time-of-flight mass spectrometer (LC/Q-TOF-MS; Agilent Technologies, USA; Metabolomics Research Center for Functional Materials, Kyungsung University). The chromatographic separation was performed on a UHPLC Agilent 1290 Infinity LC system equipped with a ZORBAX RRHD Eclipse XDB-C18 column (2.1 × 50 mm, 1.8 μm; set temperature, 30 °C; Agilent Technologies) maintained at a set temperature of 30 °C. Each sample, prepared from cellular metabolite extracts, was injected into the chromatographic system using a volume of 1 μL. The mobile phase consisted of two components: mobile phase A, comprising 45% water with 0.1% formic acid, and mobile phase B, consisting of 55% acetonitrile with 0.1% formic acid. Gradient elution was performed at a flow rate of 0.5 mL/min according to the following protocol: initial conditions of 2% B were held for 1 min, followed by a linear increase to 100% B over 8 min, maintaining 100% B for 2 min, then returning to 2% B within 1 min, and finally equilibrating at 2% B for 9 min. Mass spectrometric analysis was conducted using an Agilent 6545 Q-TOF/MS system equipped with both positive and negative electrospray ionization (ESI) sources. The instrument parameters were optimized as follows: capillary voltage of 4000 V, fragmentor voltage of 125 V, gas temperature of 300 °C, drying gas flow rate of 10 L/min, maximum nebulizer pressure of 45 psi, sheath gas temperature of 300 °C, sheath gas flow rate of 11 L/min, and RF voltage of 750 V. Data acquisition was performed using MassHunter Software 15.0, including the acquisition module version 11.0 and qualitative analysis module version 10.0 (Agilent Technologies). Full-scan mass spectra were acquired over a mass range of 100 to 1000 m/z in both positive and negative ion modes to comprehensively capture the metabolite profiles.

### 2.6. Data Processing and Analysis

The raw data files (‘-.d’) generated from LC/Q-TOF-MS were initially converted to the ‘-.cef’ format using Profinder 10.0 software (Agilent Technologies) to facilitate subsequent processing and analysis. Following conversion, the data underwent comprehensive processing steps, including peak finding, alignment, and metabolite identification, utilizing MassHunter Mass Profiler Profession 15.0 software (Agilent Technologies). This rigorous processing ensured accurate and reliable identification of metabolites from the complex mass spectrometry data. Furthermore, to gain deeper insights into the biological significance of the differentially identified metabolites, enrichment and pathway analysis were conducted using MetaboAnalyst 6.0 (http://www.metaboanalyst.ca; accessed on 10 January 2024).

### 2.7. Statistical Analysis

Data were analyzed using GraphPad Prism 9 software (USA), and the results are presented as mean ± standard error of mean (SEM) from at least three independent experiments. The normal distribution of the data was assessed using the Shapiro–Wilk test. For normally distributed data, the unpaired *t*-test was utilized to compare two groups, while one-way analysis of variance (ANOVA) was employed to compare three or more categorical groups. In instances where a significant difference was observed following one-way ANOVA, Tukey multiple comparison test was applied for post hoc analysis to determine specific group differences.

## 3. Results

### 3.1. Different Metabolomic Profile between KRAS-Wildtype and KRAS-Mutant Pancreatic Cancer Cells

**Figure 1:**
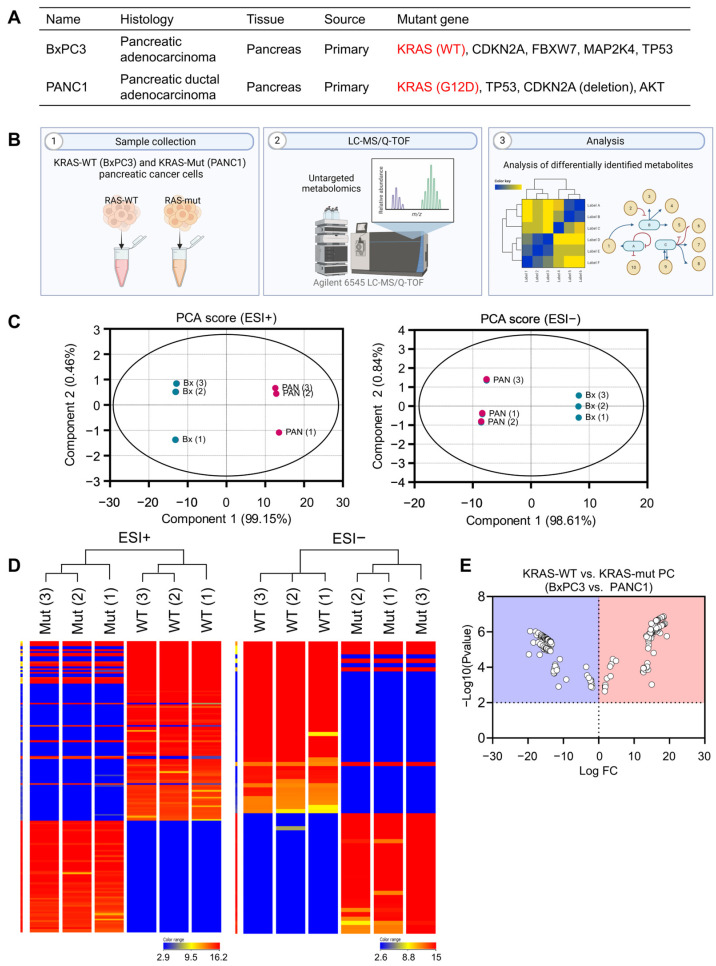
Untargeted metabolites from KRAS-WT (BxPC3) and KRAS-mutant (PANC1) pancreatic cancer cells. (**A**) Characterization of pancreatic cancer cells utilized in the present study. (**B**) A schematic diagram illustrating the untargeted metabolomics approach using UHPLC/Q-TOF-MS. (**C**) Principal component analysis (PCA) score plots for ESI-positive and -negative modes. (**D**) Hierarchical clustering trees of the samples. (**E**) A volcano plot with changes exceeding two-fold.

We investigated the cancerous features of pancreatic cancer cells based on their KRAS genetic status, utilizing both KRAS-wildtype BxPC3 and KRAS-mutant PANC1 cell lines. Their characteristics are summarized in Figure 1A. Employing untargeted metabolomics (Figure 1B), we identified a total of 195 metabolites differentially altered by KRAS status, as detailed in Table S1. Principal component analysis (PCA) score plots for both ESI-positive and ESI-negative modes demonstrated a clear separation between BxPC3 and PANC1 cells (Figure 1C), further supported by hierarchical condition trees that effectively clustered cancer samples based on their KRAS status (Figure 1D). Notably, significantly different metabolites with alterations exceeding two-fold were visualized in a volcano plot (Figure 1E). Each point on the volcano plot diagram represents a metabolite. The horizontal axis represents multiple metabolites compared with each corresponding substance, while the vertical axis represents *t*-test values.

### 3.2. Metabolite Set Enrichment and Pathway Analysis of KRAS-Wildtype and KRAS-Mutant Pancreatic Cancer Cells

**Figure 2:**
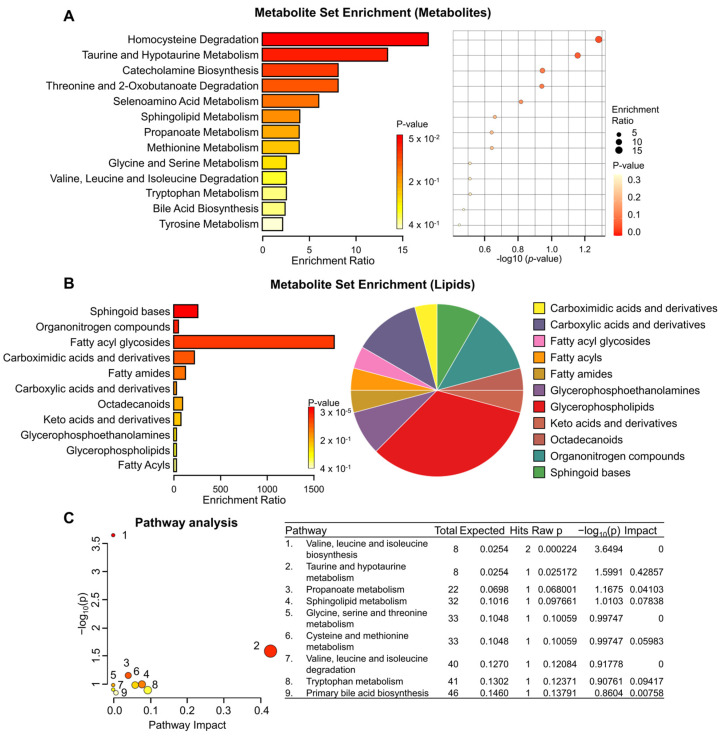
Metabolite set enrichment and pathway analysis depending on KRAS status. (**A**) Metabolite set enrichment analysis highlighting the top 13 sets. (**B**) Lipid set enrichment analysis depicting the top 11 sets. (**C**) Human KEGG pathways of the differentially identified metabolites between KRAS-WT BxPC3 and KRAS-mutant PANC1. Color indicates the levels of significance (−log_10_(*p*)) from yellow to red. The size of the circle indicates the pathway impact.

To elucidate biologically meaningful patterns enriched in our quantitative metabolomic data, we conducted metabolite set enrichment analysis, which revealed the top 13 sets including pathways such as homocysteine degradation and taurine and hypotaurine metabolism (Figure 2A). Additionally, lipid enrichment analysis highlighted 11 enriched sets, encompassing fatty acyl glycosides and sphingoid bases, among others, and a pie chart providing a graphical depiction of the distribution of enriched lipid classes is shown in Figure 2B. Furthermore, to elucidate the functional roles of the identified metabolites, we mapped them to KEGG pathways, leading to the identification of nine significant metabolic pathways associated with KRAS status, as illustrated in Figure 2C. These pathways include valine, leucine, and isoleucine biosynthesis; propanoate metabolism; and primary bile acid biosynthesis, among others. Overall, our findings underscore the distinct metabolite changes induced by KRAS status, shedding light on the diverse metabolic pathways implicated in pancreatic cancer cells.

### 3.3. Alterations in Metabolomic Profile of KRAS-Mutant Pancreatic Cancer Cells by TRPML1 Inhibition

**Figure 3:**
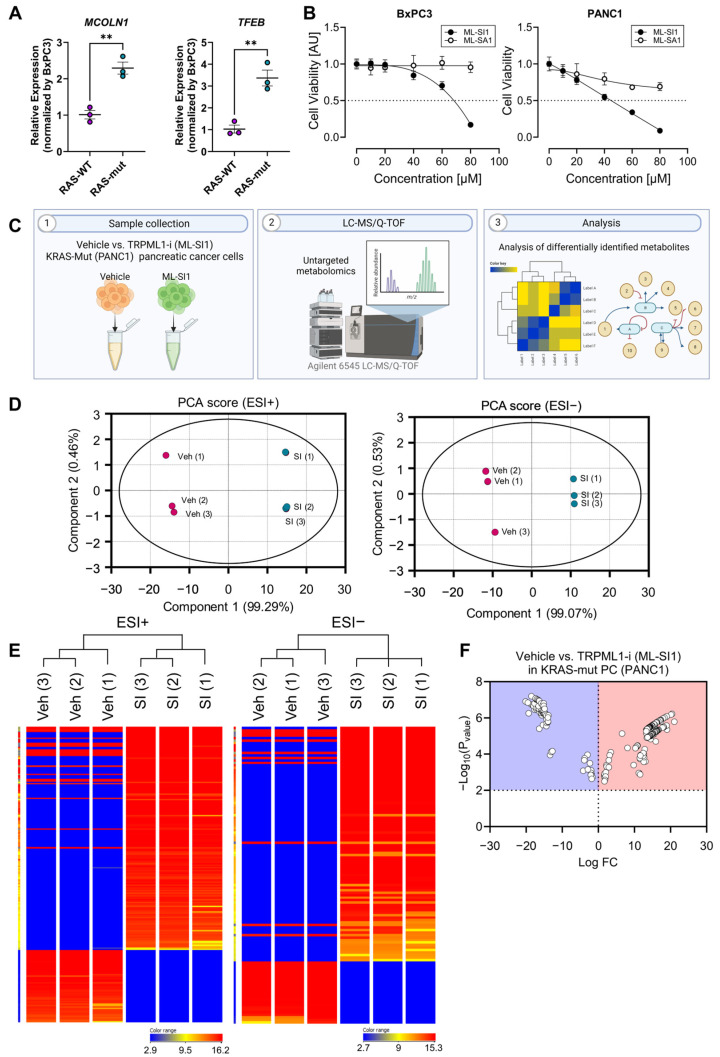
Untargeted metabolomic profile identified from TRPML1 inhibition in KRAS-mutant PANC1 cells. (**A**) Comparison of gene expressions (*MCOLN1* and *TFEB*) involved in lysosomal biogenesis and function between BxPC3 and PANC1. (**B**) The cell viability of BxPC3 and PANC1 cells following exposure to ML-SI1 or ML-SA1 for 48 h. (**C**) A schematic diagram presenting the untargeted metabolomic approach using UHPLC/Q-TOF-MS. (**D**) PCA score plots for ESI-positive and -negative modes. (**E**) Hierarchical clustering trees of the samples. (**F**) A volcano plot with changes exceeding two-fold. Data are presented as mean ± SEM (** *p* < 0.01).

Pancreatic cancer cells harboring oncogenic RAS mutations exhibit an elevated level of *MCOLN1*, encoding the lysosomal calcium channel TRPML1 [20]. TRPML1 inhibition leads to cholesterol misplacement from the cellular membrane to lysosomes, causing the removal of oncogenic HRAS from the cell surface, a reduction in downstream signaling, and eventual induction of cell death [21]. Consistent with previous studies, KRAS-mutant PANC1 displayed notable upregulation of genes such as *MCOLN1* and *TFEB*, associated with lysosomal biogenesis and function, compared to KRAS-WT BxPC3 cells. (Figure 3A). Pharmacological inhibition of TRPML1 using ML-SI1 significantly decreased PANC1 cell viability, whereas pharmacological activation of TRPML1 using ML-SA1 failed to significantly affect cell viability as observed with ML-SI1 (Figure 3B). The sensitivity to TRPML1 inhibition was higher in KRAS-mutant PANC1 cells than KRAS-WT BxPC3 cells (Figure 3B). To identify significantly different metabolites in response to 48 h exposure to ML-SI1 in KRAS-mutant PANC1 cells, untargeted metabolomic analysis was conducted as illustrated in Figure 3C. A total of 292 metabolites were found to be differentially altered by TRPML1 inhibition, as detailed in Table S2. PCA score plots for both ESI-positive and ESI-negative modes demonstrated a distinct separation between vehicle-treated and ML-SI1-treated PANC1 cells (Figure 3D), which was further supported by hierarchical condition trees effectively clustering cancer samples in response to TRPML1 inhibition (Figure 3E). Remarkably, metabolites exhibiting alterations exceeding two-fold were prominently depicted in a volcano plot (Figure 3F). Each point on the volcano plot diagram represents a metabolite. The horizontal axis represents multiple metabolites compared with each corresponding substance, while the vertical axis represents *t*-test values.

### 3.4. Metabolite Set Enrichment and Pathway Analysis of KRAS-mutant Pancreatic Cancer Cells by TRPML1 Inhibition

**Figure 4:**
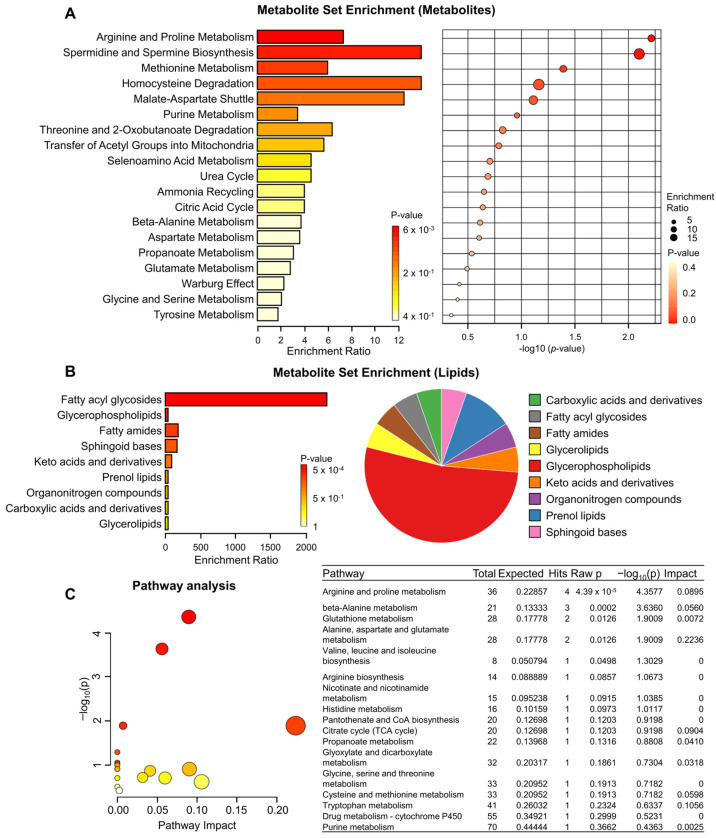
Metabolomic analyses revealing alterations induced by TRPML1 inhibition in KRAS-mutant PANC1 cells. (**A**) Metabolite set enrichment analysis highlighting the top 19 sets. (**B**) Lipid set enrichment analysis depicting the top 9 sets. (**C**) Human KEGG pathways of the differentially identified metabolites between vehicle- and ML-SI1-treated KRAS-mutant PANC1 cells. Color indicates the levels of significance (−log_10_(*p*)) from yellow to red. The size of the circle indicates the pathway impact.

To identify biologically significant patterns enriched in our quantitative metabolomic data, we conducted metabolite set enrichment analysis, revealing the top 19 sets, comprising pathways such as arginine and proline metabolism, spermidine and spermine biosynthesis, homocysteine degradation, and the malate–aspartate shuttle (Figure 4A). Additionally, lipid enrichment analysis identified nine enriched sets, which included fatty acyl glycosides, and a pie chart offering a visual representation of the distribution of enriched lipid classes is shown in Figure 4B. Furthermore, to elucidate the functional roles of the identified metabolites, we mapped them to KEGG pathways, leading to the identification of 17 significant metabolic pathways associated with TRPML1 inhibition, as depicted in Figure 4C. These pathways encompassed arginine and proline metabolism, beta-alanine metabolism, glutathione metabolism, and alanine, aspartate, and glutamate metabolism, among others. Of particular interest, when we compared alterations in metabolites commonly identified in our present two untargeted metabolomic analyses, we found that some metabolites identified in PANC1 compared to BxPC3 were oppositely regulated by TRPML1 inhibition (Table 1). This discovery suggests that these metabolites could serve as potential biomarkers detectable in KRAS-mutant cancer cells. In summary, our findings highlight the discernible changes in metabolite profiles induced by TRPML1 inhibition using ML-SI1, offering insights into the diverse metabolic pathways affecting KRAS-mutant pancreatic cancer cells. 

## 4. Discussion

In this study, untargeted metabolomics was employed to uncover potential biomarkers to distinguish between KRAS-WT and KRAS-mutant pancreatic cancer cells. The metabolomic profiles of both pancreatic cancer cell lines were distinctly characterized by their KRAS genetic status. Notably, KRAS-mutant PANC1 cells exhibited enrichment in metabolites linked to homocysteine degradation, taurine and hypotaurine metabolism, and fatty acyl glycosides. Furthermore, we validated that inhibiting the lysosomal calcium channel TRPML1 effectively induced cell death in KRAS-mutant pancreatic cancer cells. Simultaneously, we identified the metabolites affected by TRPML1 inhibition in PANC1 cells. The metabolites differentially identified following TRPML1 inhibition in KRAS-mutant PANC1 cells revealed involvement in KEGG metabolic and signaling pathways related to arginine and proline metabolism, the malate–aspartate shuttle, homocysteine degradation, and fatty acyl glycosides. Interestingly, metabolites associated with homocysteine degradation and fatty acyl glycosides were consistently detected in both untargeted metabolomic analyses.

TRPML1 has recently emerged as a promising target for tackling oncogenic autophagy in pancreatic cancer [22]. It primarily operates within lysosomes, facilitating the efflux of cations into the cytosol [23]. Some studies have explored the involvement of TRPML1 in cancer progression and resistance to chemotherapy [24,25]. Recent insights suggest that, in addition to its conventional role in maintaining lysosomal function and balance, TRPML1 aids in communication between lysosomes and mitochondria to regulate mitochondrial calcium levels, integrates cellular stress signals with lysosomal biogenesis, modulates membrane lipid assembly to enhance oncogenic signaling, and promotes the release of extracellular ATP to support invasive cancer behavior [21,26,27,28]. Notably, increased TRPML1 expression has been associated with cancer development [21,24,25,27,28,29]. Jung et al. revealed a correlation between HRAS-driven cancers and elevated TRPML1 expression [21,30]. Higher TRPML1 expression is associated with poorer patient prognosis in head and neck squamous cell carcinoma and bladder urothelial carcinoma characterized by frequent HRAS mutation [21,30]. Studies have shown that increased TRPML1 expression and activity in HRAS-driven cancer cells are crucial for cholesterol localization in the cell membrane, while TRPML1 inhibition reduces ERK phosphorylation and cell proliferation. [21,30]. Comparisons between cancer cells carrying HRAS wildtype and those with oncogenic HRAS mutations have highlighted the vulnerability of oncogenic HRAS-driven cancer cells to genetic and pharmacological TRPML1 inhibition, indicating TRPML1 as a potential target for HRAS-driven cancers [21,30]. Clinically, TRPML1 expression is elevated in various malignancies. In non-small-cell lung cancer, high TRPML1 expression correlates with advanced tumor stages and is positively associated with tumor development including proliferation, migration, and invasion. [29]. Similarly, increased TRPML1 expression in PDAC inversely correlates with patient survival rates and recurrence-free survival [27]. Patients with PDAC with worse prognosis tend to exhibit higher TRPML1 levels [27]. Given the pivotal role of autophagy in PDAC pathogenesis, regulated by TRPML1 and TFEB, TRPML1 knockdown impedes PDAC cell proliferation in vitro and suppresses tumor formation and growth in vivo [27,31,32], Unlike HRAS-driven cancer, the precise anticancer mechanisms of TRPML1 inhibition are not clearly specified in KRAS-driven pancreatic cancer. In this context, our untargeted metabolomic analysis aims to elucidate its molecular mechanisms. 

Chemotherapy remains a cornerstone in the treatment of pancreatic cancer, primarily due to the fact that more than 80% of patients are deemed inoperable upon diagnosis [33]. Currently, the combination of gemcitabine and erlotinib stands as the standard treatment for inoperable or post-surgery patients, yet secondary treatment options are limited when these drugs fail [34,35]. Therefore, potential therapeutic targets should be discovered for new treatment strategies for gemcitabine-resistant pancreatic cancer. Earlier research has indicated that BxPC3 cells exhibit heightened sensitivity to gemcitabine, whereas PANC1 cells demonstrate pronounced resistance to the drug [36,37]. Notably, despite this difference, both cell lines possess some common genetic traits such as mutations in CDKN2A, MAPD2K4, and TP53 [38]. Hence, we opted for these two cell lines and performed an extensive metabolomics analysis to explore potential therapeutic markers of gemcitabine resistance. From the metabolomics analysis of BxPC3 and PANC1 cells following treatment with the TRPML1 inhibitor (40 µM of ML-SI1), we identified 151 differentially enriched metabolites. Medina et al. reported that UV exposure or BH-mimetic treatment induced high levels of spermidine in apoptotic Jurkat cells and creatine in A549 lung cancer and HCT-116 colon cancer cells, which serve as metabolite secretomes of apoptosis [39]. Similarly, our results also demonstrate that TRPML1 inhibition leads to higher levels of creatine and lower levels of spermidine in PANC1 compared to BxPC3 (Table S3). This implies that TRPML1 inhibitor can induce apoptosis in both cancer cell lines irrespective of resistance status. Furthermore, TRPML1 inhibitor-treated BxPC3 and PANC1 cells exhibited opposing levels of L-aspartic acid, citric acid, and fumaric acid, which are associated with alanine, aspartate, and glutamate metabolism. Interestingly, a previous study by Garcia-Bermudez et al. reported that inhibition of the electron transport chain (ETC) results in a significant decrease in aspartate levels in sensitive cancer cell lines, whereas the levels of aspartate are largely maintained in resistant cancer cell lines [40]. In our current dataset, the level of L-aspartic acid was relatively lower in BxPC3 cells. This observation suggests that TRPML1 inhibition induces ETC-mediated apoptotic cell death in BxPC3 cells but not in PANC1 cells. 

A recent study identified 23 distinct metabolites from KRAS-mutant human PDAC cells and mouse acinar cells harboring the oncogenic KRAS mutation through single-cell MS analysis [41]. These findings indicated high abundance in valine, spermine, spermidine, creatine, and more. Similarly, our research detected distinct metabolites involved in various amino acid metabolic pathways, encompassing valine, leucine, and isoleucine biosynthesis and taurine and hypotaurine metabolism, methionine metabolism, glycine and serine metabolism, arginine and proline metabolism, and aspartate metabolism in PANC1 compared to BxPC3. Notably, in response to TRPML1 inhibition, we observed a low abundance of 2-ketobutyric acid particularly elevated in KRAS-mutant PANC1 cells compared to KRAS-WT BxPC3 cells. Also known as α-ketobutyrate or 2-oxobutyrate, 2-ketobutyric acid is a byproduct of amino acid metabolism, including glycine, methionine, valine, leucine, serine, threonine, and isoleucine [42,43,44]. Among those amino acids, 2-ketobutyric acid can be produced from L-methionine through the catalysis of methionine γ-lyase (MGL). Methionine, an essential amino acid, assumes crucial roles in polyamine formation and serves as a precursor for DNA and protein methylation [45]. The imposition of L-methionine restriction demonstrates potent antitumor effects on cancers reliant on this amino acid, such as breast, prostate, and lung cancers [46,47,48,49]. Normal cells can recycle methionine via re-methylation of homocysteine, catalyzed by methionine synthase or betaine–homocysteine methyltransferase [50,51]. However, cancer cells often exhibit heightened methionine synthase activity and are more vulnerable to its inhibition compared to normal tissues [52,53,54]. The substitution of methionine with homocysteine effectively hampers the proliferation of various cancer cell lines, including leukemia, breast, lung, kidney, prostate, and colon cancer [55,56,57]. Recent findings by He et al. highlighted frequent low expression levels of methionine sulfoxide reductase A (MSRA), a reducing enzyme of oxidized methionine residue, in metastatic tumor tissues of patients with PDAC. Decreased MSRA levels sustain methionine oxidation, which, in turn, supports the activity of pyruvate kinase M2 (PKM2), promoting the respiration, migration, and metastasis of PDAC [58]. In addition, a previous study demonstrated that the introduction of mutant KRAS (G12D substitution) into BxPC3 resulted in an increase in CTH compared to KRAS-WT, leading to increased ROS generation and glycolysis [59]. In our study, α-ketobutyric acid, which can be derived from thionine by CTH, also exhibited significant differences and showed potential as a biomarker. Similarly, α-ketobutyric acid was found to be higher in PANC1 with mutant KRAS (G12D) compared to KRAS-WT BxPC3, and the level of α-ketobutyric acid decreased when cell death was induced in PANC1. These results indicate the potential of α-ketobutyric acid as a biomarker or therapeutic target in pancreatic cancer harboring KRAS G12D mutation. 

Although we observed alterations in the specific metabolites, such as 2-ketobutyric acid, Eicosanoyl-EA, PE(19:1(9Z)/0:0), PI(20:4(5Z,8Z,11Z,14Z)/0:0), LysoPE(0:0/20:0), and more, in response to TRPML1 inhibition, further elucidation is needed to clarify the molecular mechanisms underlying the interaction of these metabolites with TRPMl1 and KRAS mutation. Moreover, given that our study is restricted to analyses in specific cancer cell lines, rather than patient-derived cancer cells or mouse models, it is difficult to perfectly capture the information of actual patients with PDAC, such as oncogenic profiles or the degree of tumor heterogeneity. Nevertheless, if we apply the current findings to patient samples or a human pancreatic cancer organoid system, we may expect outcomes that are more pragmatic and pertinent to the clinical setting.

## 5. Conclusions

Our findings underscore the potential of metabolomics in unraveling intricate metabolic pathways implicated in pancreatic cancer. The identification of dysregulated metabolites, such as 2-ketobutyric acid, sheds light on novel therapeutic targets and diagnostic markers for this disease. Further exploration of these metabolic alterations may pave the way for personalized treatment strategies and improved patient outcomes. Additionally, elucidating the role of methionine metabolism and its associated enzymes in tumor progression highlights promising avenues for therapeutic intervention. Ultimately, our study contributes to the growing body of evidence supporting the pivotal role of metabolism in cancer biology and underscores the importance of comprehensive metabolic profiling in understanding and combatting pancreatic cancer. Considering tumor heterogeneity, the tumor microenvironment, and the multiple actions of other oncogenes alongside KRAS mutation, applying our metabolomic profiles to an organoid system established from patients with pancreatic cancer, rather than a two-dimensional in vitro culture system, may offer greater clinical utilities. 

## Figures and Tables

**Table 1:** Alterations in common metabolites by TRPML1 inhibition.

Compound	BxPC3 vs. PANC1		Veh vs. ML-SI1 in PANC1	
	Log FC	P (corr)	Log FC	P (corr)
2-Ketobutyric acid	17.191	3.20 × 10^−5^	−17.387	1.72 × 10^−5^
Eicosanoyl-EA	14.908	1.38 × 10^−4^	−15.064	9.04 × 10^−5^
PE(19:1(9Z)/0:0)	−14.896	1.46 × 10^−4^	2.085	2.53 × 10^−2^
PI(20:4(5Z,8Z,11Z,14Z)/0:0)	−14.625	3.64 × 10^−4^	11.528	3.03 × 10^−2^
PS(21:0/20:5(5Z,8Z,11Z,14Z,17Z))	−14.342	3.64 × 10^−4^	12.680	2.22 × 10^−3^
(4E,8E,10E-d18:3)sphingosine	−14.531	1.44 × 10^−4^	14.428	1.25 × 10^−4^
4,4′-Biphenyldithiol	−17.092	1.71 × 10^−4^	15.919	5.51 × 10^−4^
LysoPE(0:0/20:0)	−13.412	2.20 × 10^−4^	16.017	1.24 × 10^−4^

## Data Availability

The raw data supporting the conclusions of this article will be made available by the authors on request.

## Supplementary Materials

The following supporting information can be downloaded at: https://www.mdpi.com/article/10.3390/biomedicines12040865/s1, Table S1: Metabolites differentially identified in KRAS-WT and KRAS-mutant pancreatic cancer cells (BxPC3 vs. PANC1); Table S2: Metabolites differentially identified in the treatment groups of vehicle and ML-SI1 (vehicle- vs. ML-SI1-treated); Table S3: Metabolites differentially identified in KRAS-WT BxPC3 and KRAS-mutant PANC1 following treatment of ML-SI1 for 48 h (BxPC3 vs. PANC1).
