# Supplementary material for: Metabolomic Approach to Identify Potential Biomarkers in KRAS-Mutant Pancreatic Cancer Cells
Source: Biomedicines. 2024 Apr 15;12(4):865. doi: 10.3390/biomedicines12040865 (PMC11048406; doi:10.3390/biomedicines12040865)
Supplement: Supplementary file 1 [file biomedicines-12-00865-s001.zip › Table S1. All entities_WT vs Mut PC.pdf]

Table S1. Metabolites differentially identified in KRAS-WT and KRAS-mutant pancreatic cancer cells (BxPC3 vs. PANC1).

Compound	Log FC	p	p (Corr)	Regulation
Erythrose	-19.753	1.86x10^-5	6.90x10^-4	down
C7 H15 N5 O3	-19.377	9.02x10^-7	1.38x10^-4	down
PE(O-16:0/17:0)	-18.449	3.99x10^-6	1.71x10^-4	down
C5 H7 N6 O2	-17.843	9.58x10^-7	1.38x10^-4	down
PE(9:0/9:0)[U]	-17.536	1.51x10^-6	1.38x10^-4	down
4,4'-Biphenyldithiol	-17.092	3.95x10^-6	1.71x10^-4	down
Kynurenine	-17.077	1.16x10^-6	1.38x10^-4	down
Bufexamac	-16.812	1.44x10^-6	1.38x10^-4	down
Pinacidil	-16.675	1.96x10^-5	7.21x10^-4	down
C3 H5 N4 O2	-16.639	2.10x10^-6	1.38x10^-4	down
C8 H8 N3 O9	-16.516	4.99x10^-6	3.48x10^-4	down
Levuglandin E2	-16.377	5.49x10^-6	3.49x10^-4	down
C11 H26 N O2 S	-16.356	2.11x10^-6	1.38x10^-4	down
C13 H18 N13 O6	-16.313	1.43x10^-6	1.38x10^-4	down
PE(9:0/9:0)[U] Esi+10.643333	-16.228	1.86x10^-6	1.38x10^-4	down
C37 H39 N8	-16.167	1.69x10^-6	1.38x10^-4	down
Sphinganine	-16.125	1.72x10^-6	1.38x10^-4	down
C11 H17 N7 O9 S	-16.015	1.60x10^-6	1.38x10^-4	down
Stearamide	-15.945	1.59x10^-6	1.38x10^-4	down
C19 H41 N O2	-15.831	1.72x10^-6	1.38x10^-4	down
C12 H23 N O5	-15.826	3.26x10^-6	1.59x10^-4	down
N-(2,3-Dihydroxybenzoyl)-L-serine	-15.784	6.17x10^-6	3.64x10^-4	down
N-Oleoyl-L-Serine	-15.680	1.74x10^-6	1.38x10^-4	down
C27 H49 O18 S	-15.599	2.18x10^-6	1.38x10^-4	down
C46 H51 N O	-15.507	9.02x10^-6	3.64x10^-4	down
C17 H23 N6 O	-15.477	9.57x10^-6	3.64x10^-4	down
Spisulosine	-15.435	2.08x10^-6	1.38x10^-4	down
C23 H19 O6 S	-15.406	1.90x10^-6	1.38x10^-4	down
Levuglandin E2 Esi-4.733333	-15.352	7.42x10^-6	3.64x10^-4	down
Hectochlorin	-15.306	2.00x10^-6	1.38x10^-4	down
C22 H24 N3	-15.252	3.99x10^-6	1.71x10^-4	down
Spisulosine Esi+5.2253337	-15.187	2.30x10^-6	1.41x10^-4	down
C12 H21 N O4	-15.177	2.16x10^-6	1.38x10^-4	down
C14 N O S	-15.092	2.08x10^-6	1.38x10^-4	down
PS(18:0/20:3(8Z,11Z,14Z))	-15.075	5.36x10^-6	2.15x10^-4	down
PS(12:0/12:0)[U]	-15.021	2.38x10^-6	1.43x10^-4	down
PE(P-18:0/17:1(9Z))	-15.021	2.10x10^-6	1.38x10^-4	down
S-(1,2-Dicarboxyethyl)Glutathione	-15.017	8.54x10^-6	3.64x10^-4	down
13,14-dihydro-15-keto Prostaglandin J2	-15.015	2.17x10^-6	1.38x10^-4	down
PE(13:0/10:0)[U]	-14.998	2.50x10^-6	1.43x10^-4	down
C8 H17 N5 O4	-14.930	2.50x10^-6	1.43x10^-4	down
C27 H43 N8 O10 S	-14.916	8.18x10^-6	3.64x10^-4	down
C7 H13 N O2 S	-14.901	2.56x10^-6	1.44x10^-4	down
C17 H26 N3 O19	-14.897	8.16x10^-6	3.64x10^-4	down

PE(19:1(9Z)/0:0)	-14.896	2.77x10^-6	1.46x10^-4	down
C10 H15 O10 S2	-14.864	2.22x10^-6	1.39x10^-4	down
13,14-dihydro-15-keto Prostaglandin J2 Esi+4.403333	-14.807	2.89x10^-6	1.49x10^-4	down
Glycerophospho-N-Oleoyl Ethanolamine Esi-5.9833336	-14.763	9.14x10^-6	3.64x10^-4	down
L 735821	-14.743	8.63x10^-6	3.64x10^-4	down
PI(18:1(9Z)/0:0)	-14.743	3.32x10^-6	1.59x10^-4	down
C38 H67 N9 O4	-14.742	4.94x10^-6	1.99x10^-4	down
C31 H43 N17	-14.738	8.68x10^-6	3.64x10^-4	down
C12 H15 N O2 S	-14.638	2.51x10^-6	1.43x10^-4	down
PI(20:4(5Z,8Z,11Z,14Z)/0:0)	-14.625	8.94x10^-6	3.64x10^-4	down
C17 H38 N15 O8	-14.605	2.46x10^-6	1.43x10^-4	down
ADP-Mannose	-14.596	1.12x10^-5	3.97x10^-4	down
N-palmitoyl serine	-14.581	2.64x10^-6	1.44x10^-4	down
(4E,8E,10E-d18:3)sphingosine	-14.531	2.68x10^-6	1.44x10^-4	down
PI(16:1(9Z)/0:0)	-14.502	9.29x10^-6	3.64x10^-4	down
1S,9R-Hydrastine	-14.439	2.97x10^-6	1.52x10^-4	down
Lauryl hydrogen sulfate	-14.375	1.04x10^-5	3.82x10^-4	down
LysoPE(0:0/22:4(7Z,10Z,13Z,16Z))	-14.361	2.66x10^-6	1.44x10^-4	down
PS(21:0/20:5(5Z,8Z,11Z,14Z,17Z))	-14.342	9.70x10^-6	3.64x10^-4	down
C15 H33 N O	-14.329	2.76x10^-6	1.46x10^-4	down
?-Tocotrienol	-14.322	9.71x10^-6	3.64x10^-4	down
C24 H41 N8	-14.314	3.57x10^-6	1.62x10^-4	down
5-Oxoavermectin "2a" aglycone	-14.196	2.84x10^-6	1.48x10^-4	down
C23 H47 N11	-14.080	9.45x10^-6	3.61x10^-4	down
C26 H20 N12 O12	-13.910	1.14x10^-5	3.97x10^-4	down
N-stearoyl glutamic acid	-13.870	4.22x10^-6	1.79x10^-4	down
Cysteineglutathione disulfide	-13.860	3.34x10^-6	1.59x10^-4	down
Lauroyl-EA	-13.832	3.39x10^-6	1.59x10^-4	down
PE(18:1(11Z)/18:3(9Z,12Z,15Z))	-13.706	1.01x10^-5	3.79x10^-4	down
C11 H19 N O2	-13.702	3.38x10^-6	1.59x10^-4	down
PE(O-18:1(9Z)/0:0)	-13.678	1.21x10^-5	4.02x10^-4	down
?-Tocotrienol Esi-8.610001	-13.658	1.21x10^-5	4.02x10^-4	down
C16 H33 N O4	-13.632	3.65x10^-6	1.62x10^-4	down
20,21,21-Trifluoro-3-methoxy-19-nor-17alpha-pregna-1,3,5(10),20-tetraen-17-ol	-13.586	3.63x10^-6	1.62x10^-4	down
C41 H63 N10 O	-13.580	3.76x10^-6	1.65x10^-4	down
C29 H31 N8 O3	-13.571	4.70x10^-6	1.92x10^-4	down
C47 H67 N4	-13.568	3.45x10^-6	1.60x10^-4	down
LysoPE(0:0/20:0)	-13.412	5.59x10^-6	2.20x10^-4	down
C8 H20 N12 O2	-13.260	4.67x10^-6	1.92x10^-4	down
C13 H29 N8 O2	-12.756	1.67x10^-4	5.64x10^-3	down
C26 H33 N10 O2	-12.671	2.14x10^-4	7.02x10^-3	down
C43 H53 N6 O2	-12.582	2.22x10^-4	7.22x10^-3	down
C30 H47 N10 O3	-12.548	1.13x10^-4	3.94x10^-3	down
PS(22:2(13Z,16Z)/18:2(9Z,12Z))	-12.333	7.09x10^-5	2.56x10^-3	down
TG(16:1(9Z)/16:1(9Z)/18:2(9Z,12Z))[iso3]	-12.317	8.49x10^-5	3.04x10^-3	down
Nafronyl	-11.891	1.13x10^-4	3.94x10^-3	down

C29 H44 N21	-11.860	1.46x10^-4	3.75x10^-3	down
C42 H67 N2 O2	-11.353	1.22x10^-3	3.69x10^-2	down
C29 H35 N7 O3	-10.972	3.51x10^-5	1.10x10^-3	down
C33 H14 N5	-9.199	4.92x10^-4	1.16x10^-2	down
C25 H47 N O4	-3.546	9.41x10^-5	3.32x10^-3	down
PE(18:0/15:0)	-3.284	2.87x10^-4	9.22x10^-3	down
PI(18:1(9Z)/0:0)	-2.807	3.93x10^-4	9.58x10^-3	down
C11 H15 N O10	-2.353	5.72x10^-4	1.33x10^-2	down
C23 H45 N O4	-2.283	6.10x10^-4	1.90x10^-2	down
C19 H24 N3 O13	-2.185	7.90x10^-4	2.44x10^-2	down
PI(18:0/0:0)	-2.172	1.25x10^-3	2.77x10^-2	down
C8 H11 N5 O9	-2.136	5.32x10^-4	1.68x10^-2	down
C17 Sphinganine	-2.105	6.06x10^-4	1.90x10^-2	down
C30 H49 O7	-1.899	1.00x10^-3	3.06x10^-2	down
5-Oxoavermectin "1a" aglycone	-1.845	1.38x10^-3	4.10x10^-2	down
C13 H5 N3 O	1.706	2.29x10^-3	4.92x10^-2	up
C9 H11 O4	1.784	1.40x10^-3	4.12x10^-2	up
C7 H5 N6 O2	1.791	1.13x10^-3	3.44x10^-2	up
C4 H6 N6 O4	1.877	9.86x10^-4	3.03x10^-2	up
C30 H53 O6	1.896	1.30x10^-3	3.86x10^-2	up
C28 H49 O6	2.311	4.28x10^-4	1.36x10^-2	up
C12 H13 N3 O5	3.226	8.99x10^-5	3.19x10^-3	up
C4 H Cl3 N2 O5 S	3.347	3.16x10^-4	7.85x10^-3	up
C7 H19 N3 O5 S	3.548	6.64x10^-5	2.41x10^-3	up
C8 H19 N O6 S	3.974	3.46x10^-5	1.27x10^-3	up
Dehydroascorbic acid	4.890	4.31x10^-5	1.31x10^-3	up
PG(22:0/18:1(9Z))	12.513	1.73x10^-4	5.82x10^-3	up
C39 H48 N9 O	12.638	1.90x10^-4	6.32x10^-3	up
7-keto-stearic acid	12.651	1.12x10^-4	3.04x10^-3	up
C32 H19 N4 O15	12.721	1.08x10^-4	2.98x10^-3	up
C26 H43 N2	13.166	1.40x10^-4	4.76x10^-3	up
C22 H40 N24 O2	13.191	1.35x10^-4	4.66x10^-3	up
C28 H55 N5 O3	13.456	1.70x10^-6	1.38x10^-4	up
C16 H25 N4 O	13.503	1.89x10^-6	1.38x10^-4	up
PS(20:4(5Z,8Z,11Z,14Z)/19:0)	13.538	1.06x10^-4	2.98x10^-3	up
C30 H49 N7 O3	13.666	9.26x10^-5	2.71x10^-3	up
PI(19:1(9Z)/13:0)	13.734	5.90x10^-5	1.76x10^-3	up
C29 H43 N10 O4	13.949	1.34x10^-4	3.52x10^-3	up
C31 H41 N12	13.997	8.55x10^-6	3.32x10^-4	up
C20 H42 N2 O2	14.063	2.17x10^-6	1.38x10^-4	up
C50 H82 N3	14.072	3.38x10^-6	1.59x10^-4	up
1-Linoleoyl Glycerol	14.078	2.60x10^-6	1.44x10^-4	up
C39 H70 N10 O3	14.125	1.37x10^-6	1.38x10^-4	up
C48 H53 N4	14.150	5.59x10^-6	2.20x10^-4	up
C23 H41 N3 O3	14.177	2.17x10^-6	1.38x10^-4	up
C27 H50 N13 O4	14.207	2.13x10^-6	1.38x10^-4	up
PI(16:0/20:5(5Z,8Z,11Z,14Z,17Z))	14.418	1.25x10^-6	1.38x10^-4	up
C34 H43 N11 O	14.437	2.14x10^-6	1.38x10^-4	up

PE(17:0/0:0)	14.480	1.36x10^-6	1.38x10^-4	up
MID42466:11?-(4-dimethylaminophenyl)-1?,25-dihydroxyvitamin D3 / 11?-(4-dimethylaminophenyl)-1?,25-d	14.541	1.19x10^-6	1.38x10^-4	up
C38 H62 N4	14.563	1.11x10^-6	1.38x10^-4	up
C33 H20 N9 O12	14.671	7.27x10^-7	5.95x10^-5	up
C22 H15 N6 O18	14.689	1.24x10^-6	1.38x10^-4	up
C16 H20 N7 O13 Esi+0.48499998	14.778	1.02x10^-6	1.38x10^-4	up
C19 H41 N	14.785	1.54x10^-6	1.38x10^-4	up
C35 H45 N11	14.808	2.26x10^-6	1.40x10^-4	up
4-phenyl-5-methyl-1,2,3-Thiadiazole	14.872	1.00x10^-6	1.38x10^-4	up
C26 H9 N9 O15	14.880	3.41x10^-7	3.84x10^-5	up
Eicosanoyl-EA	14.908	9.98x10^-7	1.38x10^-4	up
C29 H45 N7 O	14.924	1.00x10^-6	1.38x10^-4	up
PE(13:0/13:0)	14.935	1.19x10^-6	1.38x10^-4	up
PS(21:0/22:6(4Z,7Z,10Z,13Z,16Z,19Z))	14.992	9.46x10^-4	2.13x10^-2	up
C17 H24 N5 O12	15.023	1.09x10^-6	1.38x10^-4	up
C31 H29 N4 O6	15.070	1.48x10^-6	1.38x10^-4	up
C8 N2 O4 S3	15.131	9.78x10^-7	1.38x10^-4	up
3-O-Methylisoproterenol Sulfate	15.168	4.70x10^-7	4.59x10^-5	up
C17 H24 N8 O4	15.172	1.00x10^-6	1.38x10^-4	up
C17 H18 N12 O6 S	15.190	4.04x10^-7	4.23x10^-5	up
(Z)-N-(2-hydroxyethyl)hexadec-7-enamide	15.207	9.44x10^-7	1.38x10^-4	up
C11 H13 N8 O2	15.372	9.84x10^-7	1.38x10^-4	up
C42 H66 N10 O	15.503	1.55x10^-5	5.79x10^-4	up
C9 H8 N O	15.656	1.00x10^-6	1.38x10^-4	up
C8 H17 O9	15.658	9.30x10^-7	1.38x10^-4	up
C27 H45 O4	15.660	7.97x10^-7	1.38x10^-4	up
C12 H20 N3 O11	15.665	7.90x10^-7	1.38x10^-4	up
C18 H32 N4 O4	15.733	7.79x10^-7	1.38x10^-4	up
C18 H29 O15	15.807	8.64x10^-7	1.38x10^-4	up
C21 H20 N4 O11	15.962	2.88x10^-7	3.52x10^-5	up
C6 H9 N2 O5	16.085	7.97x10^-7	1.38x10^-4	up
C11 H7 O12	16.214	2.26x10^-7	3.20x10^-5	up
C7 H13 N5 O8	16.239	2.28x10^-7	3.20x10^-5	up
Lys-Trp-OH	16.241	6.52x10^-7	1.38x10^-4	up
C9 H17 N5 O8 S	16.274	5.94x10^-7	5.44x10^-5	up
C10 H8 N6 S	16.293	7.19x10^-7	1.38x10^-4	up
Pyrroline hydroxycarboxylic acid Esi-0.33866668	16.496	2.23x10^-7	3.20x10^-5	up
C10 H7 N3	16.498	9.27x10^-7	1.38x10^-4	up
C11 H14 N2 O7	16.560	2.40x10^-7	3.20x10^-5	up
Propionylglycine	16.612	2.14x10^-7	3.20x10^-5	up
C11 H15 O6	16.772	1.97x10^-7	3.20x10^-5	up
C36 H78 N7 O4 S	16.862	7.39x10^-7	1.38x10^-4	up
C12 H3 N3 O2	16.951	5.35x10^-7	1.38x10^-4	up
N-HFG	16.954	5.65x10^-7	1.38x10^-4	up
2-Ketobutyric acid	17.191	1.76x10^-7	3.20x10^-5	up
C37 H38 O3	17.334	1.15x10^-6	1.38x10^-4	up

C3 H7 N3 O S	17.801	4.43x10^-7	1.38x10^-4	up
C8 H15 N5 O5 S	17.867	1.46x10^-7	3.20x10^-5	up
Glutarylglycine	17.944	1.46x10^-7	3.20x10^-5	up
C10 H19 N O4	17.974	4.05x10^-7	1.38x10^-4	up
C4 H2 O8 S2	18.086	3.94x10^-7	1.38x10^-4	up
Taurine	18.304	1.47x10^-7	3.20x10^-5	up
5-Hydroxy-2,4-dioxopentanoate	18.314	1.33x10^-7	3.20x10^-5	up
Tuberonic acid glucoside	18.427	3.61x10^-7	1.38x10^-4	up
1-(10-methyl-hexadecanoyl)-2-(8-[3]-ladderane-octanyl)-sn-glycerophosphoethanolamine	18.586	3.61x10^-7	1.38x10^-4	up
C8 H7 N2	18.707	3.41x10^-7	1.38x10^-4	up
L-Isoleucine	19.256	1.74x10^-6	1.38x10^-4	up
