# Supplementary material for: Metabolomic Approach to Identify Potential Biomarkers in KRAS-Mutant Pancreatic Cancer Cells
Source: Biomedicines. 2024 Apr 15;12(4):865. doi: 10.3390/biomedicines12040865 (PMC11048406; doi:10.3390/biomedicines12040865)
Supplement: Supplementary file 1 [file biomedicines-12-00865-s001.zip › Table S2. All entities_SI treated PANC1.pdf]

Table S2. Metabolites differentially identified in the treatment groups of vehicle and ML-SI1 (vehicle vs. ML-SI1-treated).

Compound	Log FC	p	p (Corr)	Regulation
C8 H19 N O6 S	-20.141	1.23x10^-7	9.04x10^-5	down
C23 H2 N2 O20 S	-19.532	1.43x10^-7	1.72x10^-5	down
C4 N O4 S	-19.280	5.50x10^-7	9.04x10^-5	down
Tuberonic acid glucoside	-18.569	1.79x10^-7	9.04x10^-5	down
5-Hydroxy-2,4-dioxopentanoate	-18.526	6.33x10^-8	1.72x10^-5	down
C10 H19 N O4	-18.135	2.01x10^-7	9.04x10^-5	down
C3 H7 N3 O S	-17.952	2.21x10^-7	9.04x10^-5	down
C39 H74 N10 O4	-17.639	3.48x10^-7	9.04x10^-5	down
CDP-DG(16:0/16:0) Esi-7.596333	-17.634	1.57x10^-7	1.72x10^-5	down
2-Ketobutyric acid	-17.387	8.40x10^-8	1.72x10^-5	down
C5 H5 N4 O3	-17.239	2.69x10^-7	9.04x10^-5	down
2-Furoic acid	-17.195	9.58x10^-8	1.72x10^-5	down
Spermidine	-17.192	3.30x10^-7	9.04x10^-5	down
C11 H15 O6	-17.000	9.31x10^-8	1.72x10^-5	down
C9 H5 N3 O7	-16.973	1.00x10^-7	1.72x10^-5	down
C7 H5 N O7	-16.756	2.93x10^-7	9.04x10^-5	down
cyclic adenosine diphosphate ribose Esi-0.365	-16.704	1.01x10^-7	1.72x10^-5	down
C22 H17 N9 O18 S	-16.538	2.20x10^-7	1.96x10^-5	down
C7 H13 N5 O8	-16.473	1.08x10^-7	1.72x10^-5	down
Lys-Trp-OH	-16.404	3.21x10^-7	9.04x10^-5	down
C34 H21 N O16	-16.363	1.15x10^-7	1.72x10^-5	down
C32 H59 N17 O3	-16.362	3.96x10^-6	1.30x10^-4	down
C33 H21 O19	-16.335	1.18x10^-7	1.72x10^-5	down
S-(Hydroxyphenylacetothiohydroximoyl)-L-cysteine	-16.309	4.53x10^-7	9.04x10^-5	down
C6 H8 Cl	-15.875	4.45x10^-7	9.04x10^-5	down
C27 H45 O4	-15.815	3.98x10^-7	9.04x10^-5	down
C26 H13 N2 O14	-15.811	1.59x10^-7	1.72x10^-5	down
C9 H8 N O	-15.810	5.38x10^-7	9.04x10^-5	down
C7 H14 N9 O	-15.758	6.42x10^-7	9.04x10^-5	down
C6 H13 N4 O	-15.648	7.03x10^-7	9.04x10^-5	down
Dihydroceramide C2	-15.630	4.95x10^-7	9.04x10^-5	down
C24 H31 N8 O	-15.498	4.84x10^-7	9.04x10^-5	down
C17 H18 N12 O6 S	-15.425	2.01x10^-7	1.96x10^-5	down
C27 H41 N7 O	-15.403	4.49x10^-7	9.04x10^-5	down
3-O-Methylisoproterenol Sulfate	-15.401	2.40x10^-7	2.01x10^-5	down
(Z)-N-(2-hydroxyethyl)hexadec-7-enamide	-15.361	4.72x10^-7	9.04x10^-5	down
PG(20:0/18:0)	-15.317	2.46x10^-6	1.24x10^-4	down
C6 H8 N O4	-15.301	3.47x10^-7	2.50x10^-5	down
PE(O-18:1(9Z)/0:0)	-15.238	4.95x10^-7	9.04x10^-5	down

C17 H24 N5 O12	-15.176	5.60x10^-7	9.04x10^-5	down
PE(P-20:0/0:0)	-15.123	2.17x10^-7	1.96x10^-5	down
C26 H9 N9 O15	-15.122	1.58x10^-7	1.72x10^-5	down
Eicosanoyl-EA	-15.064	4.95x10^-7	9.04x10^-5	down
C22 H19 N12 O13 S2	-15.041	2.98x10^-7	2.37x10^-5	down
C17 H27 N5	-15.000	5.29x10^-7	9.04x10^-5	down
C13 H27 N O	-14.972	6.01x10^-7	9.04x10^-5	down
C16 H20 N7 O13 Esi+0.48499998	-14.935	4.99x10^-7	9.04x10^-5	down
C30 H47 N7 O3	-14.884	3.15x10^-7	2.38x10^-5	down
C41 H79 N5 O3	-14.793	6.26x10^-7	9.04x10^-5	down
MID42466:11?-(4-dimethylaminophenyl)-1?,25-dihydroxyvitamin D3 / 11?-(4-dimethylaminophenyl)-1?,25-d	-14.708	5.92x10^-7	9.04x10^-5	down
C28 H49 N8 O3	-14.553	5.89x10^-7	9.04x10^-5	down
C27 H52 N O	-14.392	7.36x10^-7	9.04x10^-5	down
PS(15:1(9Z)/14:0)	-14.382	2.84x10^-6	1.24x10^-4	down
C23 H41 N3 O3	-14.361	1.17x10^-6	1.18x10^-4	down
C39 H70 N10 O3	-14.307	6.76x10^-7	9.04x10^-5	down
C16 H27 N	-14.287	1.86x10^-6	1.24x10^-4	down
C15 H25 N O	-13.965	9.49x10^-7	1.03x10^-4	down
C15 H20 N7	-13.928	1.59x10^-6	1.24x10^-4	down
C29 H53 N15 O2	-13.920	1.68x10^-6	1.24x10^-4	down
C39 H53 N6	-13.805	7.18x10^-7	9.04x10^-5	down
C26 H43 N2	-13.361	1.12x10^-4	3.02x10^-3	down
C32 H19 N4 O15	-13.036	7.48x10^-5	1.24x10^-3	down
C8 H11 N S4	-12.720	6.86x10^-5	1.15x10^-3	down
Spermine	-4.191	8.25x10^-4	2.08x10^-2	down
Costatol	-3.886	2.16x10^-4	3.17x10^-3	down
Citric acid	-3.780	1.70x10^-4	2.54x10^-3	down
C7 H15 N O3	-2.986	1.08x10^-4	2.93x10^-3	down
C33 H20 N9 O12	-2.872	6.92x10^-4	9.34x10^-3	down
C9 H15 N2 O7 S	-2.333	3.17x10^-4	8.18x10^-3	down
C10 H9 N8 O5	-2.083	1.42x10^-3	1.87x10^-2	down
Phosphocreatine	-1.990	1.22x10^-3	2.95x10^-2	down
C13 H20 N3 O6	-1.968	1.09x10^-3	2.69x10^-2	down
C7 H19 N3 O5 S	-1.946	7.93x10^-4	2.00x10^-2	down
C12 H13 N3 O5	-1.874	1.28x10^-3	3.07x10^-2	down
C16 H20 N7 O13	-1.849	1.43x10^-3	3.37x10^-2	down
C8 H15 N5 O5 S	-1.821	2.31x10^-3	2.99x10^-2	down
PE(P-20:0/0:0)	-1.569	1.91x10^-3	4.49x10^-2	down
C30 H49 O7	1.651	1.75x10^-3	4.12x10^-2	up
PE(18:0/0:0)	1.686	1.38x10^-3	3.27x10^-2	up
Glycerophospho-N-Oleoyl Ethanolamine	1.716	3.16x10^-3	4.01x10^-2	up
Glycerophospho-N-Oleoyl Ethanolamine	1.728	1.22x10^-3	2.95x10^-2	up
PE(18:0/0:0)	1.866	2.65x10^-3	3.39x10^-2	up

Pyrroline hydroxycarboxylic acid	1.935	2.01x10^-3	4.69x10^-2	up
PS(21:0/0:0)	2.031	1.72x10^-3	2.24x10^-2	up
PE(19:1(9Z)/0:0)	2.085	1.01x10^-3	2.53x10^-2	up
C16 H41 N16 O4	2.246	4.08x10^-4	1.05x10^-2	up
Adenosine5'-monophosphate	2.331	4.87x10^-4	1.24x10^-2	up
C32 H53 O7	2.370	5.09x10^-4	1.29x10^-2	up
PE(17:0/0:0)	2.915	2.16x10^-4	5.61x10^-3	up
C29 H49 O9	2.929	5.30x10^-4	7.35x10^-3	up
PE(19:0/0:0)	3.141	1.15x10^-4	3.08x10^-3	up
PE(21:0/0:0)	3.475	7.88x10^-5	2.16x10^-3	up
N-Carbamoyl-DL-aspartic acid	6.593	7.35x10^-6	2.08x10^-4	up
2-Hydroxy-C18-cerebroside	8.068	4.56x10^-5	1.27x10^-3	up
C34 H27 N18	9.794	4.03x10^-4	5.65x10^-3	up
C49 H33 N2 O2	10.285	4.71x10^-5	8.00x10^-4	up
C27 H13 N10 O14	10.464	4.09x10^-5	7.03x10^-4	up
C41 H41 N10 O2	10.584	3.94x10^-5	6.85x10^-4	up
PI(22:4(7Z,10Z,13Z,16Z)/0:0)	10.603	3.92x10^-5	6.85x10^-4	up
C51 H37 N2 O2	10.637	3.82x10^-5	6.80x10^-4	up
C18 H10 N12 O10	10.669	3.80x10^-5	6.80x10^-4	up
C22 H37 N12 O4	10.796	3.44x10^-5	6.26x10^-4	up
C34 H35 N15 O4	10.871	3.33x10^-5	6.15x10^-4	up
PS(22:2(13Z,16Z)/18:1(9Z))	11.203	1.10x10^-3	2.71x10^-2	up
PS(18:0/20:0)	11.367	1.12x10^-3	2.73x10^-2	up
C30 H46 N8 O2	11.489	1.66x10^-4	2.50x10^-3	up
PI(20:4(5Z,8Z,11Z,14Z)/0:0)	11.528	1.26x10^-3	3.03x10^-2	up
PE(18:0/0:0) Esi-5.9736667	11.564	1.83x10^-4	2.71x10^-3	up
Gibberellin A15	11.579	1.32x10^-3	3.14x10^-2	up
C30 H29 N18 O3	11.657	1.19x10^-4	1.88x10^-3	up
C26 H20 N12 O12	11.691	1.12x10^-4	1.78x10^-3	up
C37 H35 N8 O3	11.695	1.40x10^-4	2.16x10^-3	up
PS(20:4(5Z,8Z,11Z,14Z)/19:0)	12.296	1.13x10^-4	3.03x10^-3	up
C19 H36 N15 O8	12.386	1.52x10^-4	4.02x10^-3	up
C26 H35 N19 O4	12.454	8.58x10^-5	1.41x10^-3	up
C29 H35 N14 O2	12.461	1.23x10^-4	1.92x10^-3	up
C21 H18 N8	12.585	2.64x10^-4	3.81x10^-3	up
C24 H4 N4 O8	12.604	1.01x10^-4	1.63x10^-3	up
PS(21:0/20:5(5Z,8Z,11Z,14Z,17Z))	12.680	1.45x10^-4	2.22x10^-3	up
Khayasin	12.861	5.33x10^-5	1.47x10^-3	up
Tamarixetin 5-glucoside-7-glucuronide	13.061	2.54x10^-4	3.69x10^-3	up
2-oxo-nonadecanoic acid	13.276	5.42x10^-6	1.58x10^-4	up
Erythrodiol	13.286	4.55x10^-6	1.38x10^-4	up
LysoPE(20:1(11Z)/0:0)	13.301	4.60x10^-6	1.38x10^-4	up
C25 H19 N17 O3	13.312	7.22x10^-6	2.06x10^-4	up
Erythrodiol Esi+8.927667	13.331	4.59x10^-6	1.38x10^-4	up
PI(20:3(8Z,11Z,14Z)/0:0)	13.441	1.26x10^-5	2.40x10^-4	up
LysoPE(0:0/22:0) Esi+6.8786664	13.479	4.39x10^-6	1.36x10^-4	up

C32 H53 N O2	13.542	5.66x10^-6	1.64x10^-4	up
C17 H29 N17 O5	13.553	4.12x10^-6	1.32x10^-4	up
C39 H35 N5 O2	13.556	4.64x10^-6	1.38x10^-4	up
C28 H51 N7 O2	13.591	4.23x10^-6	1.33x10^-4	up
C46 H76 N4 O3	13.591	3.97x10^-6	1.30x10^-4	up
C19 H14 N8 O15	13.636	1.17x10^-5	2.30x10^-4	up
C31 H21 N14 O2	13.647	4.32x10^-6	1.34x10^-4	up
C37 H31 N11 O	13.672	4.18x10^-6	1.32x10^-4	up
C18 H4 N O21 S2	13.683	4.67x10^-6	1.39x10^-4	up
C20 H20 N3 O23	13.692	1.17x10^-5	2.30x10^-4	up
C11 H14 O17	13.699	1.20x10^-5	2.32x10^-4	up
C26 H40 N11 O	13.708	1.14x10^-5	2.30x10^-4	up
C23 H21 N	13.744	3.70x10^-6	1.28x10^-4	up
C40 H48 O3	13.818	3.85x10^-6	1.30x10^-4	up
C34 H33 N12 O	13.819	3.90x10^-6	1.30x10^-4	up
Lys Phe Lys	13.845	3.57x10^-6	1.26x10^-4	up
C38 H21 N8 O2	13.854	3.80x10^-6	1.30x10^-4	up
24-isopropenyl-22E-dehydrocholesterol Esi+8.146999	13.855	3.70x10^-6	1.28x10^-4	up
Glycerophospho-N-Oleoyl Ethanolamine Esi-5.9839997	13.902	1.09x10^-5	2.26x10^-4	up
C25 H21 N20 O8	13.914	3.91x10^-6	1.30x10^-4	up
C29 H41 N13 O4	13.915	1.09x10^-5	2.26x10^-4	up
p-HydroxyPiroxicam glucuronide	13.915	1.08x10^-5	2.26x10^-4	up
C27 H42 N8 O5	13.918	1.06x10^-5	2.26x10^-4	up
C13 H18 N6 O7	13.926	3.72x10^-6	1.28x10^-4	up
PE(O-18:0/0:0)	13.942	3.55x10^-6	1.26x10^-4	up
C18 H26 N6 O18	13.973	1.04x10^-5	2.26x10^-4	up
C35 H37 N4 O8	13.992	1.06x10^-5	2.26x10^-4	up
C8 H11 N6 O6	13.997	1.06x10^-5	2.26x10^-4	up
C28 H35 N3 O3	14.044	3.48x10^-6	1.26x10^-4	up
C31 H43 N4 O3	14.081	3.36x10^-6	1.25x10^-4	up
C30 H33 N10	14.082	3.40x10^-6	1.25x10^-4	up
C20 H27 N20 O3	14.084	3.59x10^-6	1.26x10^-4	up
C36 H35 N11 O3	14.084	1.01x10^-5	2.26x10^-4	up
C38 H34 N6 O	14.152	3.52x10^-6	1.26x10^-4	up
2-Hydroxyfelbamate	14.181	3.19x10^-6	1.25x10^-4	up
PG(16:0/0:0)[U] Esi-7.0033336	14.194	9.64x10^-6	2.26x10^-4	up
C33 H9 N10 O11	14.211	9.63x10^-6	2.26x10^-4	up
PE(18:0/0:0) Esi+5.653667	14.215	3.40x10^-6	1.25x10^-4	up
C4 H Cl2 O2 S2	14.230	9.70x10^-6	2.26x10^-4	up
C25 H45 N11 O3	14.232	3.29x10^-6	1.25x10^-4	up
C15 H10 N5 O14 S	14.236	9.84x10^-6	2.26x10^-4	up
C23 H9 N6 O13	14.236	9.69x10^-6	2.26x10^-4	up
C15 H12 N5 O4 S2	14.241	9.63x10^-6	2.26x10^-4	up
24-isopropenyl-22E-dehydrocholesterol	14.244	3.58x10^-6	1.26x10^-4	up

C21 H10 O22	14.262	9.57x10^-6	2.26x10^-4	up
LysoPE(0:0/20:0) Esi+6.183	14.268	3.27x10^-6	1.25x10^-4	up
C18 H35 N22	14.276	9.41x10^-6	2.26x10^-4	up
C13 H13 N O9	14.291	3.33x10^-6	1.25x10^-4	up
Riboflavin cyclic-4',5'-phosphate	14.299	3.25x10^-6	1.25x10^-4	up
C26 H21 N17 O3	14.317	3.22x10^-6	1.25x10^-4	up
LysoPE(0:0/22:1(13Z))	14.327	3.16x10^-6	1.25x10^-4	up
C32 H25 N14 O4	14.378	3.09x10^-6	1.25x10^-4	up
Anandamide (20:2, n-6)	14.401	6.43x10^-6	1.84x10^-4	up
C24 H16 N6 O17 S	14.405	9.46x10^-6	2.26x10^-4	up
C16 H45 N15 O2 S2	14.422	3.02x10^-6	1.25x10^-4	up
(4E,8E,10E-d18:3)sphingosine	14.428	3.00x10^-6	1.25x10^-4	up
Procaterol	14.428	4.08x10^-6	1.32x10^-4	up
PG(16:0/0:0)[U]	14.444	9.97x10^-6	2.26x10^-4	up
C16 H22 N2 O4	14.449	3.15x10^-6	1.25x10^-4	up
C8 H17 N4 O7	14.467	1.49x10^-5	4.19x10^-4	up
C20 H5 N3 O	14.497	2.96x10^-6	1.25x10^-4	up
Inosine Esi-0.7266667	14.513	9.94x10^-6	2.26x10^-4	up
C15 H16 N9 Esi+2.8553333	14.538	3.23x10^-6	1.25x10^-4	up
PG(18:1(9E)/0:0)[U]	14.548	8.91x10^-6	2.26x10^-4	up
C23 H35 O8	14.599	2.88x10^-6	1.24x10^-4	up
C10 N O6	14.602	2.86x10^-6	1.24x10^-4	up
C13 H17 N O4 S	14.623	2.81x10^-6	1.24x10^-4	up
C16 H16 N9 O8 Esi+3.78	14.628	2.88x10^-6	1.24x10^-4	up
LysoPE(0:0/22:0)	14.651	2.85x10^-6	1.24x10^-4	up
C22 H23 N2 O24	14.658	8.42x10^-6	2.26x10^-4	up
PI(20:4(5Z,8Z,11Z,14Z)/0:0) Esi-6.0156665	14.669	8.45x10^-6	2.26x10^-4	up
Inosine	14.710	8.60x10^-6	2.26x10^-4	up
PE(19:1(9Z)/0:0) Esi+5.4406667	14.741	2.80x10^-6	1.24x10^-4	up
C16 H41 N11 O9	14.741	8.11x10^-6	2.26x10^-4	up
1H-Indole-4-acetic acid, 2,3-dihydro-2-oxo-	14.811	2.72x10^-6	1.24x10^-4	up
C33 H19 N18 O Esi+5.120333	14.811	2.68x10^-6	1.24x10^-4	up
C35 H52 N4 O3	14.821	3.18x10^-6	1.25x10^-4	up
C15 H16 N9	14.822	2.74x10^-6	1.24x10^-4	up
L-Formylkynurenine	14.844	2.61x10^-6	1.24x10^-4	up
3'-Methoxy-E,E-dienoestrol	14.846	2.58x10^-6	1.24x10^-4	up
C31 H49 N7 O2	14.875	2.77x10^-6	1.24x10^-4	up
C26 H14 N2 O8	14.933	2.63x10^-6	1.24x10^-4	up
C19 H12 N8 O8	14.939	2.59x10^-6	1.24x10^-4	up
4-Methylumbelliferyl sulfate	14.963	7.72x10^-6	2.26x10^-4	up
Amaronol B	15.009	8.73x10^-6	2.26x10^-4	up
C43 H51 N8 O	15.011	2.83x10^-6	1.24x10^-4	up
C29 H45 N7 O4	15.024	7.44x10^-6	2.26x10^-4	up
C5 H8 N O9	15.036	7.55x10^-6	2.26x10^-4	up
C30 H12 N4 O2	15.045	2.63x10^-6	1.24x10^-4	up

C24 H14 N7 O6	15.078	2.48x10^-6	1.24x10^-4	up
2-Amino-3,7-dideoxy-D-threo-hept-6-ulosonic acid	15.083	7.36x10^-6	2.26x10^-4	up
C30 H53 N O2	15.117	2.53x10^-6	1.24x10^-4	up
C14 H14 N9 O8	15.167	2.40x10^-6	1.24x10^-4	up
Dihydrocordoin	15.170	2.45x10^-6	1.24x10^-4	up
7-Hydroxypipotiazine glucuronide	15.238	2.32x10^-6	1.24x10^-4	up
4-Heptyloxyphenol	15.265	2.32x10^-6	1.24x10^-4	up
PE(O-16:0/0:0)	15.266	6.89x10^-6	2.26x10^-4	up
PI(20:4(5Z,8Z,11Z,14Z)/0:0)	15.281	6.86x10^-6	2.26x10^-4	up
Captopril disulfide	15.311	2.29x10^-6	1.24x10^-4	up
C27 H23 N17 O6 Esi+5.120333	15.347	2.27x10^-6	1.24x10^-4	up
C21 H23 N20 O6	15.372	2.22x10^-6	1.24x10^-4	up
Immepip	15.398	5.81x10^-6	1.67x10^-4	up
C21 H12 N9 O5	15.403	2.27x10^-6	1.24x10^-4	up
n-Pentadecylamine	15.430	2.29x10^-6	1.24x10^-4	up
C5 H5 N O5	15.445	7.84x10^-6	2.26x10^-4	up
C32 H57 N O3	15.445	2.28x10^-6	1.24x10^-4	up
C14 H14 N2 O11 S	15.451	2.25x10^-6	1.24x10^-4	up
C33 H21 N18 O2	15.466	2.15x10^-6	1.24x10^-4	up
C13 H4 O16	15.523	6.45x10^-6	2.26x10^-4	up
9-bromo-decanoic acid	15.556	2.29x10^-6	1.24x10^-4	up
C12 H2 N2 O9	15.565	6.35x10^-6	2.26x10^-4	up
C8 H4 N3 O11	15.592	2.30x10^-6	1.24x10^-4	up
C14 H16 O18	15.593	6.25x10^-6	2.26x10^-4	up
C30 H14 N4 O4	15.676	2.05x10^-6	1.24x10^-4	up
C29 H19 N19 O2	15.710	2.05x10^-6	1.24x10^-4	up
C4 H8 N3 O5	15.727	5.98x10^-6	2.26x10^-4	up
C21 H20 N2 O17	15.727	6.04x10^-6	2.26x10^-4	up
C23 H14 N8 O5	15.785	2.00x10^-6	1.24x10^-4	up
cis-12a-Hydroxyrot-2'-enonic acid	15.820	2.02x10^-6	1.24x10^-4	up
C27 H19 N21	15.900	1.93x10^-6	1.24x10^-4	up
Met-Tyr-OH	15.905	1.93x10^-6	1.24x10^-4	up
4,4'-Biphenyldithiol	15.919	1.98x10^-5	5.51x10^-4	up
C27 H23 N17 O4	15.940	1.90x10^-6	1.24x10^-4	up
C29 H14 N3 O4	15.963	1.93x10^-6	1.24x10^-4	up
C12 H14 N O14	15.965	5.64x10^-6	2.26x10^-4	up
Maleamic acid	15.974	4.11x10^-6	1.32x10^-4	up
C42 H25 N5 O4	16.016	1.87x10^-6	1.24x10^-4	up
LysoPE(0:0/20:0)	16.017	1.88x10^-6	1.24x10^-4	up
Melphalan	16.038	1.85x10^-6	1.24x10^-4	up
CDP-DG(16:0/16:0)	16.057	5.85x10^-6	2.26x10^-4	up
C33 H19 N18 O	16.069	1.80x10^-6	1.24x10^-4	up
C25 H23 N20 O6	16.079	1.80x10^-6	1.24x10^-4	up
C10 H10 O7 S	16.100	5.35x10^-6	2.26x10^-4	up
PE(O-16:0/0:0)	16.157	1.74x10^-6	1.24x10^-4	up

C14 H4 N O13	16.196	5.20x10^-6	2.26x10^-4	up
Thr Ser Ser	16.240	1.70x10^-6	1.24x10^-4	up
C32 H57 N O2	16.262	1.71x10^-6	1.24x10^-4	up
C10 H4 O7	16.328	5.02x10^-6	2.26x10^-4	up
Gly Asp Asp	16.352	5.01x10^-6	2.26x10^-4	up
C26 H20 N O4	16.402	1.65x10^-6	1.24x10^-4	up
C16 H10 N6 O11 S	16.566	4.72x10^-6	2.26x10^-4	up
C27 H23 N17 O6	16.627	1.52x10^-6	1.24x10^-4	up
C27 H23 N11 O4	16.629	1.55x10^-6	1.24x10^-4	up
4-Methylumbelliferyl ?-D-glucuronide	16.721	4.51x10^-6	2.26x10^-4	up
C22 H12 N10 O3	16.750	1.48x10^-6	1.24x10^-4	up
C26 H22 N O5	16.934	1.42x10^-6	1.24x10^-4	up
C7 H9 Cl N2 O2 S	17.099	1.40x10^-6	1.24x10^-4	up
PE(14:0/18:0)	17.187	1.30x10^-6	1.20x10^-4	up
C16 H14 N9 O7	17.296	1.28x10^-6	1.20x10^-4	up
C20 H21 N17 O4	17.309	1.27x10^-6	1.20x10^-4	up
PE(18:0/16:1(9Z))	17.368	1.56x10^-6	1.24x10^-4	up
C16 H16 N9 O8	17.408	1.24x10^-6	1.20x10^-4	up
C5 H4 N3 O8	17.799	3.42x10^-6	1.92x10^-4	up
Chlorophacinone	17.930	1.07x10^-6	1.10x10^-4	up
C8 H4 N6 O5 S	18.047	3.18x10^-6	1.88x10^-4	up
C3 H4 N5 O3	18.154	3.24x10^-6	1.88x10^-4	up
C34 H25 N11 O3	18.352	9.60x10^-7	1.03x10^-4	up
C13 H14 N2 O11	18.901	2.66x10^-6	1.68x10^-4	up
C27 H23 N17 O3	18.908	8.37x10^-7	9.60x10^-5	up
C16 H16 N9 O9	18.958	8.23x10^-7	9.60x10^-5	up
5-Aminopentanoic acid	19.028	4.42x10^-6	1.36x10^-4	up
C5 H4 N5 O4	20.413	5.96x10^-7	9.04x10^-5	up
L-Aspartic Acid	21.042	1.63x10^-6	1.07x10^-4	up
