# Supplementary material for: Metabolomic Approach to Identify Potential Biomarkers in KRAS-Mutant Pancreatic Cancer Cells
Source: Biomedicines. 2024 Apr 15;12(4):865. doi: 10.3390/biomedicines12040865 (PMC11048406; doi:10.3390/biomedicines12040865)
Supplement: Supplementary file 1 [file biomedicines-12-00865-s001.zip › Table S3. Metabolites differentially identified.pdf]

Table S3. Metabolites differentially identified in KRAS-WT BxPC3 and KRAS-mutant PANC1 following treatment of ML-SI1 for 48 h (BxPC3 vs. PANC1).

Compound	Log FC	p (corr)	Abundance (Log_2)	
			SI-treated BxPC3	SI-treated PANC1
Creatine	2.408625	$2.49x10^{-4}$	20.19716	22.60578
L-Aspartic Acid	22.04454	$1.26x10^{-8}$	0.250326	22.29487
N-Carbamoyl-DL-aspartic acid	21.6861	$1.02x10^{-7}$	0.612549	22.29865
PE(15:0/16:0)	21.49742	$9.09x10^{-8}$	0.612549	22.10997
PE(18:0/15:1(9Z))	1.784996	$1.59x10^{-3}$	19.33322	21.11821
Pyrroline hydroxycarboxylic acid	1.320992	$2.64x10^{-3}$	19.67329	20.99428
Octylamine	20.25474	$9.02x10^{-7}$	0.612549	20.86729
2-C-Methyl-D-erythritol 4-phosphate	2.359703	$3.09x10^{-4}$	18.35122	20.71092
Pyrroline hydroxycarboxylic acid Esi-0.46333337	1.341156	$3.52x10^{-3}$	18.65091	19.99207
PE(18:0/15:1(9Z)) Esi+10.6830015	3.503849	$5.86x10^{-5}$	16.1559	19.65975
UDP-glucose	1.521963	$6.67x10^{-4}$	17.92674	19.4487
PE(14:0/22:2(13Z,16Z))	18.6895	$1.87x10^{-7}$	0.612549	19.30205
Chlorophacinone	18.60014	$1.34x10^{-7}$	0.612549	19.21269
PE(18:0/16:1(9Z))	18.04962	$2.55x10^{-7}$	0.612549	18.66217
Glutarylglycine	18.33183	$1.86x10^{-8}$	0.250326	18.58215
PE(18:0/15:0)	17.9367	$1.60x10^{-5}$	0.612549	18.54925
PE(14:0/18:0)	17.8727	$1.62x10^{-7}$	0.612549	18.48525
5-Deoxy-5-(methylthio)adenosine	2.67561	$1.66x10^{-4}$	15.7379	18.41351
PE(17:0/0:0)	2.601787	$2.48x10^{-4}$	15.67825	18.28004
4-Methylumbelliferyl-D-glucuronide	17.86235	$2.19x10^{-8}$	0.250326	18.11267
Taurine	17.70654	$2.19x10^{-8}$	0.250326	17.95686
N-HFG	17.24822	$2.01x10^{-7}$	0.612549	17.86077
PS(O-20:0/17:0)	17.5579	$2.43x10^{-8}$	0.250326	17.80822
PE(P-18:0/19:0)	17.08223	$2.68x10^{-7}$	0.612549	17.69478
PE(16:0/0:0)	1.127069	$2.50x10^{-3}$	16.39835	17.52542
PE(O-16:0/0:0)	16.85867	$2.13x10^{-7}$	0.612549	17.47122
Maleamic acid	16.67934	$9.84x10^{-7}$	0.612549	17.29189
PE(O-16:0/0:0)	16.4783	$3.17x10^{-8}$	0.250326	16.72862
Propionylglycine	16.41448	$3.26x10^{-8}$	0.250326	16.6648
2-Amino-3,7-dideoxy-D-threo-hept-6-ulosonic acid	16.28381	$3.72x10^{-8}$	0.250326	16.53413
PE(18:0/16:1(9Z)) Esi+10.711333	15.81645	$4.12x10^{-7}$	0.612549	16.429
4-Methylumbelliferyl sulfate	16.16664	$3.99x10^{-8}$	0.250326	16.41696
PE(11:0/10:0)[U]	15.74293	$3.41x10^{-7}$	0.612549	16.35548
PE(12:0/18:0)[U]	15.71828	$3.05x10^{-7}$	0.612549	16.33083
PE(15:0/16:0) Esi+10.725001	15.51078	$4.44x10^{-6}$	0.612549	16.12333
PS(21:0/15:1(9Z))	15.66979	$1.23x10^{-7}$	0.250326	15.92012
PE(22:1(11Z)/19:1(9Z))	15.08739	$4.03x10^{-7}$	0.612549	15.69994
p-HydroxyPiroxicam glucuronide	15.25595	$5.31x10^{-8}$	0.250326	15.50628
Tamarixetin 5-glucoside-7-glucuronide	15.17412	$1.06x10^{-6}$	0.250326	15.42444
PE(O-18:0/0:0)	14.71531	$4.23x10^{-7}$	0.612549	15.32786
PI(20:4(8Z,11Z,14Z,17Z)/16:0)	14.53318	$4.43x10^{-7}$	0.612549	15.14573

PS(20:4(5Z,8Z,11Z,14Z)/17:2(9Z,12Z))	14.50993	$5.21x10^{-7}$	0.612549	15.12248
PS(21:0/20:5(5Z,8Z,11Z,14Z,17Z))	14.09457	$6.95x10^{-6}$	0.988203	15.08277
2,2-Dimethyl-3-(4-methoxyphenyl)-4-propyl-2H-1-benzopyran-7-ol acetate	14.38857	$2.63x10^{-6}$	0.612549	15.00112
PS(20:1(11Z)/17:1(9Z))	14.68798	$8.03x10^{-8}$	0.250326	14.93831
PI(20:4(5Z,8Z,11Z,14Z)/18:4(6Z,9Z,12Z,15Z))	14.27554	$1.04x10^{-6}$	0.612549	14.88809
D-Glucosyldihydrosphingosine	14.26756	$7.36x10^{-7}$	0.612549	14.8801
N-cis-octadec-9Z-enoyl-L-Homoserine lactone	14.19759	$9.65x10^{-7}$	0.612549	14.81014
PS(20:4(5Z,8Z,11Z,14Z)/19:0)	13.96552	$8.17x10^{-7}$	0.612549	14.57806
2-Hydroxy-C18-cerebroside	13.44691	$7.23x10^{-7}$	0.612549	14.05946
PS(16:0/13:0)	12.09795	$7.87x10^{-4}$	0.612549	12.7105
Diglycolic acid	-1.70861	$2.83x10^{-3}$	21.63849	19.92988
N1-Acetylspermidine	-1.50728	$2.11x10^{-3}$	19.38656	17.87929
C17 Sphinganine	-1.60432	$1.72x10^{-3}$	19.15855	17.55422
PS(21:0/0:0) Esi-6.0281663	-1.56272	$8.18x10^{-4}$	18.99284	17.43012
Citric acid	-3.80753	$2.39x10^{-5}$	20.71918	16.91165
PI(18:1(9Z)/0:0) Esi-6.5464993	-2.80413	$5.35x10^{-5}$	19.69727	16.89313
PI(20:4(5Z,8Z,11Z,14Z)/0:0)	-1.1953	$2.56x10^{-3}$	17.93491	16.73961
5-Oxoavermectin "1a" aglycone	-2.26876	$3.90x10^{-4}$	18.85631	16.58755
PI(18:0/0:0)	-2.9836	$3.50x10^{-5}$	19.54138	16.55777
PI(18:1(9Z)/0:0)	-4.26093	$1.07x10^{-5}$	20.62956	16.36863
PS(18:1(9Z)/0:0)	-1.34324	$4.20x10^{-3}$	17.68472	16.34148
1-(10-methyl-hexadecanoyl)-2-(8-[3]-ladderane-octanyl)-sn-glycerophosphoethanolamine	-1.48597	$2.64x10^{-3}$	17.69868	16.21271
PG(18:1(9E)/0:0)[U]	-3.91442	$2.23x10^{-5}$	19.98584	16.07142
PS(O-16:0/19:0)	-2.75684	$1.99x10^{-4}$	18.62548	15.86864
24-isopropenyl-22E-dehydrocholesterol	-3.83406	$4.78x10^{-5}$	19.4447	15.61064
Glycerophospho-N-Oleoyl Ethanolamine Esi-5.9839997	-1.38235	$1.29x10^{-3}$	16.87592	15.49357
Erythrodiol Esi+8.9275	-3.01737	$1.11x10^{-4}$	17.80879	14.79142
Erythrodiol	-3.14917	$8.85x10^{-5}$	17.90201	14.75284
PI(22:4(7Z,10Z,13Z,16Z)/0:0)	-1.21958	$3.50x10^{-3}$	15.80057	14.58098
Glycidyl oleate	-3.48563	$3.28x10^{-4}$	17.97976	14.49413
PE(14:0/10:0)[U]	-14.2634	$3.77x10^{-5}$	16.87641	2.613026
PS(P-16:0/13:0)	-11.7901	$6.72x10^{-5}$	14.33697	2.546895
PE(O-16:0/17:0)	-17.3045	$5.91x10^{-7}$	18.48719	1.18272
PI(18:1(9Z)/0:0)	-16.8142	$7.04x10^{-7}$	17.99694	1.18272
PS(P-16:0/17:2(9Z,12Z))	-16.7023	$1.26x10^{-6}$	17.88498	1.18272
PE-NMe2(16:0/18:1(9Z))	-16.3332	$1.48x10^{-6}$	17.51594	1.18272
Spermidine	-16.3121	$8.40x10^{-7}$	17.49486	1.18272
PS(22:6(4Z,7Z,10Z,13Z,16Z,19Z)/17:2(9Z,12Z))	-16.3062	$9.40x10^{-7}$	17.48895	1.18272
Sphinganine	-16.2402	$8.19x10^{-7}$	17.42294	1.18272
PG(18:1(9E)/0:0)[U]	-16.2338	$8.67x10^{-7}$	17.41652	1.18272
PE(11:0/14:0)[U]	-16.1332	$8.35x10^{-7}$	17.31589	1.18272
3'-O-Aminopropyl-25-hydroxyvitamin D3	-15.8078	$9.46x10^{-7}$	16.99048	1.18272
PE(P-18:0/17:2(9Z,12Z))	-15.5054	$9.92x10^{-7}$	16.68812	1.18272
Guanosine	-15.5035	$1.29x10^{-6}$	16.68623	1.18272

Succinoadenosine	-15.2775	$1.11x10^{-6}$	16.46024	1.18272
Dihydroceramide C2	-15.2093	$1.22x10^{-6}$	16.39204	1.18272
PI(20:4(5Z,8Z,11Z,14Z)/0:0)	-15.1434	$1.28x10^{-6}$	16.32613	1.18272
PI(20:3(8Z,11Z,14Z)/0:0)	-15.1175	$1.24x10^{-6}$	16.30021	1.18272
PI(16:1(9Z)/0:0)	-15.0657	$1.20x10^{-6}$	16.24838	1.18272
4,4'-Diapophytofluene	-14.9216	$1.39x10^{-6}$	16.10434	1.18272
LysoPE(0:0/20:4(5Z,8Z,11Z,14Z))	-14.8136	$1.33x10^{-6}$	15.9963	1.18272
3-Hydroxy-5-chola-7,9(11)-dien-24-oic Acid	-14.8029	$1.25x10^{-6}$	15.98563	1.18272
Arachidonoylmorpholine	-14.646	$1.43x10^{-6}$	15.82872	1.18272
5beta-Pregnane-3alpha,17alpha,20alpha-triol	-14.6253	$1.39x10^{-6}$	15.80803	1.18272
PG(20:4(5Z,8Z,11Z,14Z)/0:0)	-14.5595	$1.53x10^{-6}$	15.74227	1.18272
PE(6:0/6:0)	-14.4439	$1.43x10^{-6}$	15.62666	1.18272
Butoctamide hydrogen succinate	-14.2966	$1.72x10^{-6}$	15.47934	1.18272
Dihydrojasmonic Acid, Methyl Ester	-14.144	$1.63x10_{-6}$	15.32675	1.18272
17beta-Nitro-5alpha-androstane	-14.096	$1.84x10^{-6}$	15.27868	1.18272
Oleoyl Ethanolamide	-14.0279	$1.85x10^{-6}$	15.21061	1.18272
PE(O-18:1(9Z)/0:0)	-14.0173	$1.84x10^{-6}$	15.19998	1.18272
PI(18:2(9Z,12Z)/0:0)	-14.0048	$1.73x10^{-6}$	15.1875	1.18272
cyclic adenosine diphosphate ribose	-13.944	$1.85x10^{-6}$	15.12675	1.18272
35-aminobacteriohopane-31,32,33,34-tetrol	-13.9413	$1.83x10^{-6}$	15.12398	1.18272
26,27-Dihomo-1-hydroxy-24-epivitamin D2	-13.931	$1.87x10^{-6}$	15.11367	1.18272
L-Tyrosine methyl ester 4-sulfate	-13.8462	$1.80x10^{-6}$	15.02896	1.18272
PA(16:0/13:0)	-13.8485	$1.91x10^{-6}$	15.03126	1.18272
11beta-Chloromethylestradiol	-13.7388	$1.91x10^{-6}$	14.9215	1.18272
PA(P-16:0/13:0)	-13.8537	$1.92x10^{-6}$	15.0364	1.18272
LysoPE(0:0/22:4(7Z,10Z,13Z,16Z))	-13.8845	$1.98x10^{-6}$	15.06721	1.18272
5'-CMP	-13.7921	$2.00x10^{-6}$	14.9748	1.18272
N-pentadecanoyl-L-Homoserine lactone	-13.8123	$2.03x10^{-6}$	14.99505	1.18272
PI(20:4(5Z,8Z,11Z,14Z)/0:0) Esi+6.4506664	-13.8595	$2.10x10^{-6}$	15.04221	1.18272
PE(20:4(5Z,8Z,11Z,14Z)/20:4(8Z,11Z,14Z,17Z))	-15.1223	$2.20x10^{-6}$	16.30504	1.18272
PG(20:0/18:0)	-15.0363	$2.24x10^{-6}$	16.21904	1.18272
N-Arachidonoyl-3-hydroxy-Aminobutyric Acid	-13.4948	$2.49x10^{-4}$	14.67747	1.18272
1-(O-alpha-D-glucopyranosyl)-(1,3R,29S,31R)-dotriacontanetetraol	-14.2198	$1.26x10^{-8}$	15.40248	1.18272
PS(12:0/22:0)	-13.7681	$1.02x10^{-7}$	14.9508	1.18272
Anandamide (20:2, n-6)	-13.4764	$9.09x10^{-8}$	14.65911	1.18272
PS(20:1(11Z)/0:0)	-13.5416	$1.59x10^{-3}$	14.72431	1.18272
PA(O-16:0/13:0)	-13.3924	$2.64x10^{-3}$	14.57509	1.18272
PE(P-18:0/17:1(9Z))	-14.1653	$9.02x10^{-7}$	15.34805	1.18272
cis-Caryophyllene	-13.6633	$3.09x10^{-4}$	14.84604	1.18272
PA(20:0/19:0)	-15.9172	$3.52x10^{-3}$	17.09994	1.18272
Bisabolol Acetate	-14.1936	$5.86x10^{-5}$	15.37629	1.18272
C-2 Ceramide	-12.5002	$6.67x10^{-4}$	13.68289	1.18272
Ramipril glucuronide	-11.4355	$1.87x10^{-7}$	12.61823	1.18272
TG(16:1(9Z)/16:1(9Z)/18:2(9Z,12Z))[iso3]	-11.1788	$1.34x10^{-7}$	12.36151	1.18272
PE(22:5(7Z,10Z,13Z,16Z,19Z)/P-16:0)	-11.6815	$2.55x10^{-7}$	12.86423	1.18272
PGF2 diethyl amide	-11.7179	$1.86x10^{-8}$	12.90063	1.18272

LysoPE(0:0/20:4(5Z,8Z,11Z,14Z)) Esi+6.0856667	-11.5957	$1.60x10^{-5}$	12.77847	1.18272
N-Hexadecyl-L-hydroxyproline	-11.7188	$1.62x10^{-7}$	12.90157	1.18272
3-alpha,20-alpha-dihydroxy-5-beta-pregnane 3-glucuronide	-9.65701	$1.66x10^{-4}$	10.83973	1.18272
PG(18:1(9E)/0:0)[U] Esi-7.413	-16.9111	$2.48x10^{-4}$	17.55582	0.644715
PI(18:2(9Z,12Z)/0:0)	-16.9687	$2.19x10^{-8}$	17.61337	0.644715
PI(16:1(9Z)/0:0)	-16.7752	$2.19x10^{-8}$	17.41995	0.644715
Fumaric acid	-17.4563	$2.01x10^{-7}$	18.10099	0.644715
cyclic adenosine diphosphate ribose Esi-0.364	-16.2486	$2.43x10^{-8}$	16.8933	0.644715
2-Furoic acid	-16.5087	$2.68x10^{-7}$	17.15342	0.644715
PI(18:2(9Z,12Z)/0:0) Esi-5.955667	-15.3259	$2.50x10^{-3}$	15.9706	0.644715
PG(22:6(4Z,7Z,10Z,13Z,16Z,19Z)/0:0)	-15.1333	$2.13x10^{-7}$	15.77801	0.644715
S-(1,2-Dicarboxyethyl)Glutathione	-15.3312	$9.84x10^{-7}$	15.97592	0.644715
PS(10:0/10:0)	-14.7061	$3.17x10^{-8}$	15.35079	0.644715
PI(18:0/0:0) Esi-7.2636666	-14.4583	$3.26x10^{-8}$	15.10299	0.644715
PI(20:3(8Z,11Z,14Z)/0:0) Esi-6.3836665	-14.0279	$3.72x10^{-8}$	14.6726	0.644715
Flavin adenine dinucleotide (FAD)	-14.3712	$4.12x10^{-7}$	15.01596	0.644715
PG(20:4(5Z,8Z,11Z,14Z)/0:0)	-14.0422	$3.99x10^{-8}$	14.68689	0.644715
PI(18:0/0:0) Esi-10.740334	-15.4614	$3.41x10^{-7}$	16.10608	0.644715
1,2-Distearoyl phosphatidyl serine	-15.3473	$3.05x10^{-7}$	15.99203	0.644715
PG(18:2(9Z,12Z)/0:0)	-14.2829	$4.44x10^{-6}$	14.92764	0.644715
